# Electrochemical and optoelectronic properties of terthiophene- and bithiophene-based polybenzofulvene derivatives†

**DOI:** 10.1039/c7ra13242e

**Published:** 2018-03-19

**Authors:** Fabrizia Fabrizi de Biani, Annalisa Reale, Vincenzo Razzano, Marco Paolino, Germano Giuliani, Alessandro Donati, Gianluca Giorgi, Wojciech Mróz, Daniele Piovani, Chiara Botta, Andrea Cappelli

**Affiliations:** Dipartimento di Biotecnologie, Chimica e Farmacia and European Research Centre for Drug Discovery and Development, Università degli Studi di Siena Via Aldo Moro 2 53100 Siena Italy andrea.cappelli@unisi.it +39 0577 234320; Istituto per lo Studio delle Macromolecole (CNR) Via A. Corti 12 20133 Milano Italy

## Abstract

The electrochemical behavior of some polybenzofulvene derivatives bearing bithiophene (BT) or terthiophene (TT) side chains was investigated by cyclic voltammetry. Very interestingly, the presence of unsubstituted terminal thiophene moieties allowed poly-6-BT-BF3k and poly-6-TT-BF3k to be cross-linked by electrochemical procedures. Conductive films were obtained by electrodeposition from solutions of these polymers onto electrode surfaces through the formation of covalent cross-linking due to dimerization (*i.e.* electrochemical oxidation) of the BT or TT side chains. The films showed electrochromic features and switched from yellow-orange (neutral) to green (positively charged) by switching the potential, and were stable to tenths of cycles, without degradation in the wet state in the electrolyte solution. Finally, the thin film obtained by electrodeposition of poly-6-TT-BF3k on a indium tin oxide (ITO) glass substrate showed in the neutral state a significantly red-shifted photoluminescence (PL) emission (∼40 nm red-shifted with respect to that of the corresponding film obtained by casting procedures), which was consistent with the presence of more conjugated moieties produced by the oxidative dimerization of the TT side chains. The innovative architecture and the easy preparation could lead to a broad range of applications in optoelectronics and bioelectronics for these cross-linked hybrid materials based on π-stacked polybenzofulvene backbones bearing oligothiophene side chains.

## Abstract 2

The electrochemical behavior of some polybenzofulvene derivatives bearing bithiophene (BT) or terthiophene (TT) side chains was investigated by cyclic voltammetry and cross-linked materials were obtained by dimerization of the BT or TT side chains.

## Graphical abstract

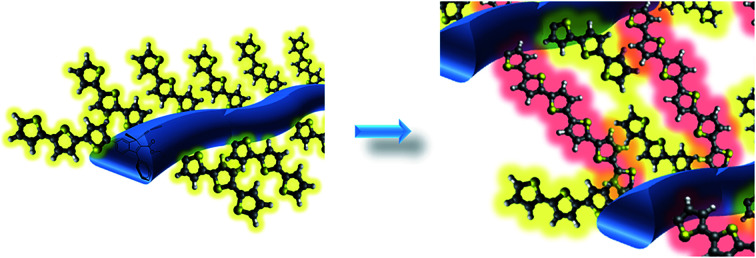

## Introduction

Polythiophene (PT) derivatives represent one of the most studied families of conducting conjugated polymers that largely contributed to the development of opto-electronic devices thanks to their outstanding charge transport properties and light-polymer responses.^1^ The most outstanding properties of these materials (*i.e.* optical absorption, electrical conductivity) are due to charge delocalization along the polymer backbone by virtue of the overlap of the π-orbitals of the thiophene monomeric units, which are required to be coplanar each other.^2^ On the other hand, the twisting of the polymer backbone interrupts conjugation and produces changes in the optoelectronic properties such as color and conductivity. Both photon absorption and chemical doping produce charged units (*i.e.* polarons and/or bipolarons), which are capable of moving by intra-chain and inter-chain hopping processes and are therefore responsible for the conducting mechanism.^2,3^ Polythiophene derivatives have been synthesized by means of standard chemical procedures or by electrochemical polymerization, by applying a potential to a solution containing both the thiophene monomer and an electrolyte to produce a conductive film on the anode (anodic route).^4^ The role of the synthesis is however fundamental in determining the type and the number of enchainment irregularities introduced during polymerization.^5,6^

On the other side, polybenzofulvene derivatives belong to a different class of conjugated polymers.^7–26^ In fact, by virtue of their π-electron systems stacked along the polymer chain,^17^ they are through-space conjugated polymers, which showed significant hole-transporting features. Moreover, the structure of benzofulvene monomeric unities were modulated in order to obtain materials for the production of optoelectronic devices such as OLEDs.^20,23^ In particular, the introduction of 9,9-dimethylfluorene (DMFL) or triphenylamine (TPA) residues in position 6 of the 3-phenylindene moieties as in poly-6-DMFL-BF3k or poly-6-TPA-BF3k, respectively, led to the development of interesting materials for optoelectronic applications.^20,23^

**Fig. 1:**
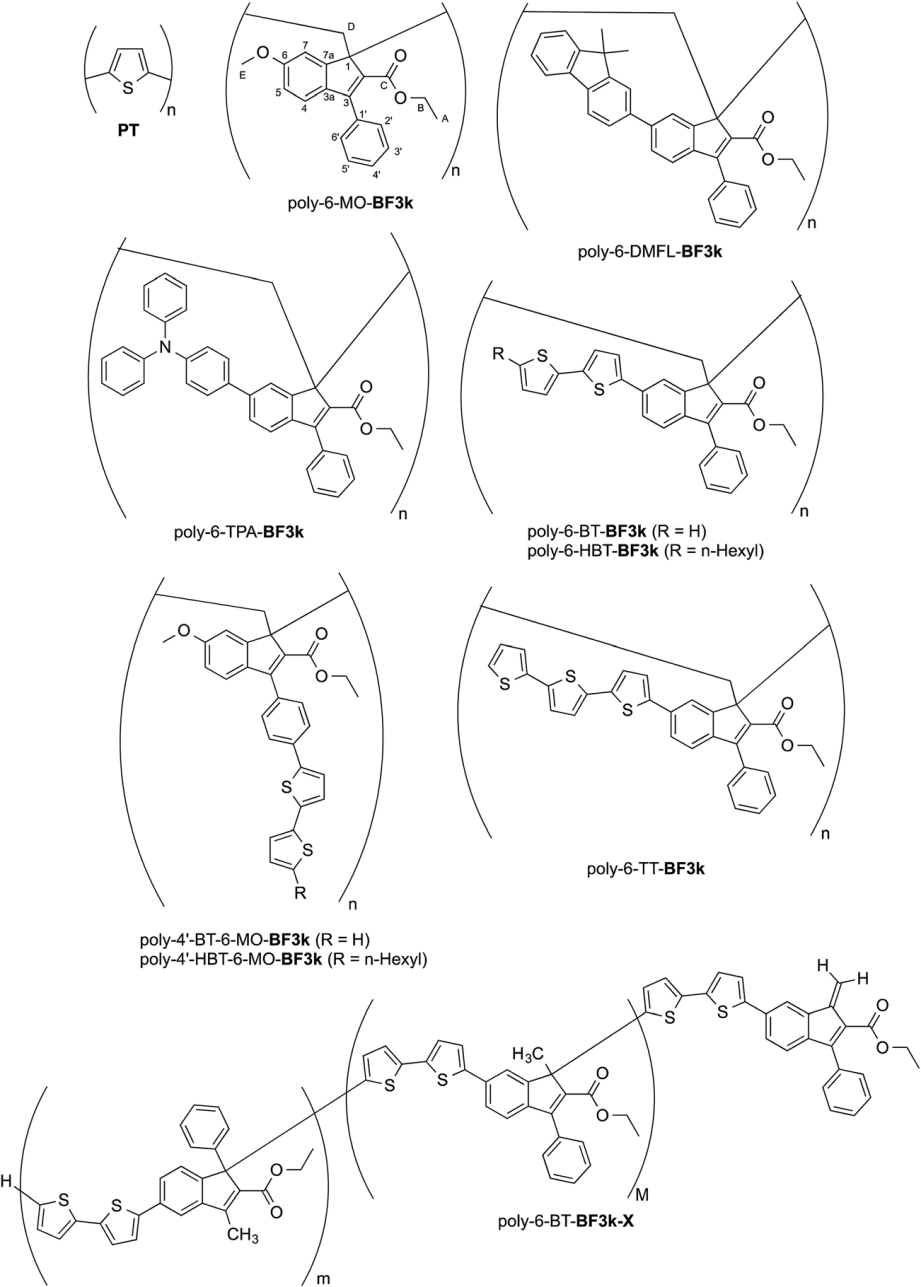
Structures of PT, poly-6-MO-BF3k, poly-6-DMFL-BF3k, poly-6-TPA-BF3k, and of polybenzofulvene derivatives bearing bithiophene or tertiophene chromophores.

The introduction of bithiophene (BT) chromophores in the two different key positions (6 and 4′) of the phenylindene scaffold produced polybenzofulvene derivatives (*i.e.* poly-6-BT-BF3k, poly-6-HBT-BF3k, poly-4′-BT-6-MO-BF3k, and poly-4′-HBT-6-MO-BF3k) showing improved hole-transporting properties and quenched emissions in the solid state that appeared to be affected by both the substitution topology of the monomeric units and the polymer enchainment (as in poly-6-BT-BF3k-X).^22^ In poly-6-BT-BF3k, poly-6-HBT-BF3k, poly-4′-BT-6-MO-BF3k, and poly-4′-HBT-6-MO-BF3k, the oligothiophene moieties are appended to the polybenzofulvene backbone as in comb-like architectures, and, in principle they should be forced to establish interchain interactions different from those observed in PT. Moreover, the presence of unsubstituted terminal thiophene moieties in poly-6-BT-BF3k and poly-4′-BT-6-MO-BF3k suggested that these polymers could be cross-linked by electrochemical procedures in order to obtain new materials showing unprecedented properties (Fig. 1).

Thus, all the members of the subfamily of polybenzofulvene derivatives bearing BT chromophores were studied in a systematic electrochemical characterization. Owing to the intriguing results obtained with poly-6-BT-BF3k (*vide infra*), the corresponding terthiophene (TT) derivative poly-6-TT-BF3k was synthesized and its electrochemical and photophysical features were compared to those of the other subfamily members.

## Results and discussion

### Synthesis and spontaneous polymerization of benzofulvene derivative bearing terthiophene chromophores 6-TT-BF3k

**Scheme 1:**
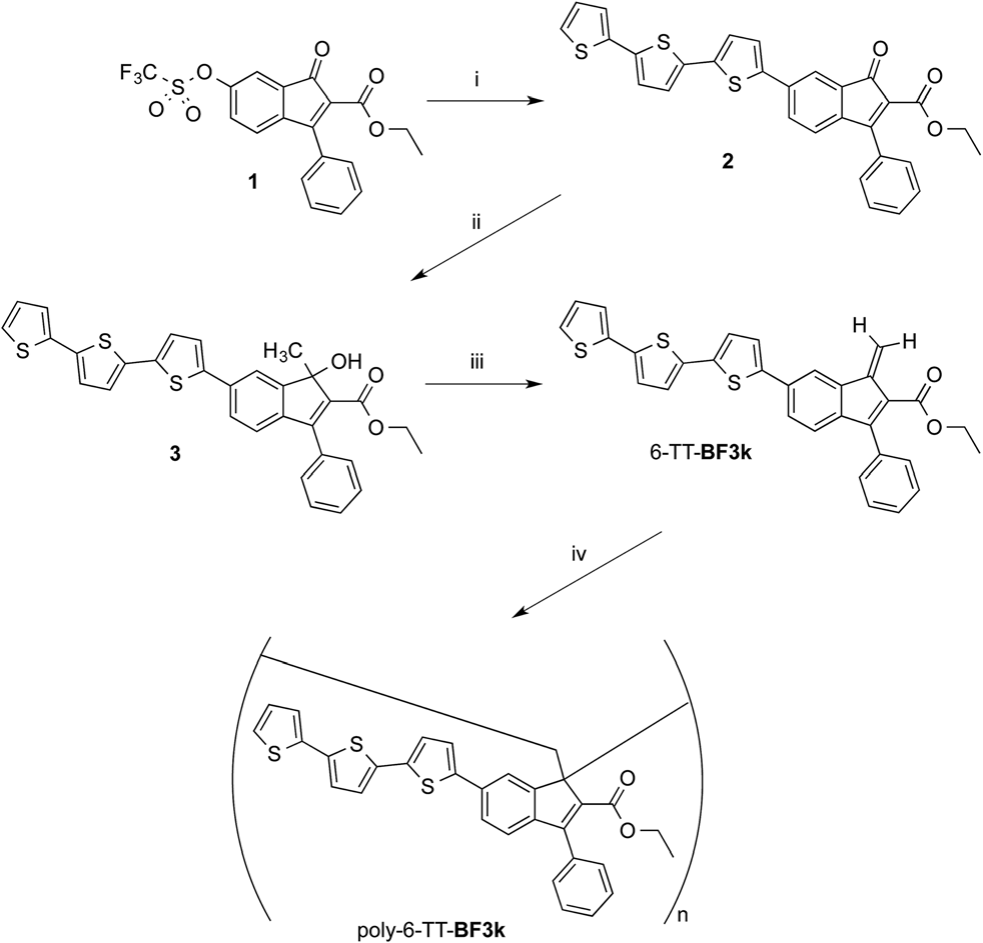
Preparation and spontaneous polymerization of benzofulvene derivative 6-TT-BF3k.

The preparation of benzofulvene derivative 6-TT-BF3k was carried out by optimizing the chemistry developed for the synthesis of the previously published members of this subfamily of polybenzofulvene derivatives.^22^ Thus, triflate 1 (ref. 18) (Scheme 1) was used as the starting material in Suzuki–Miyaura cross-coupling with 2,2′:5′,2′′-terthiophene-5-boronic acid pinacol ester (commercially available from Aldrich) to obtain indenone derivative 2 in good (*i.e.* 60%) yield.

#### Reagents

(i) 2,2′:5′,2′′-Terthiophene-5-boronic acid pinacol ester, Pd(PPh_3_)_2_Cl_2_, PPh_3_, K_3_PO_4_, 2,6-di-*tert*-butyl-4-methylphenol, C_2_H_5_OH, 1,4-dioxane; (ii) Al(CH_3_)_3_, CH_2_Cl_2_; (iii) PTSA, 2,6-di-*tert*-butyl-4-methylphenol, CHCl_3_ (or CDCl_3_) (iv) solvent evaporation .

**Fig. 2:**
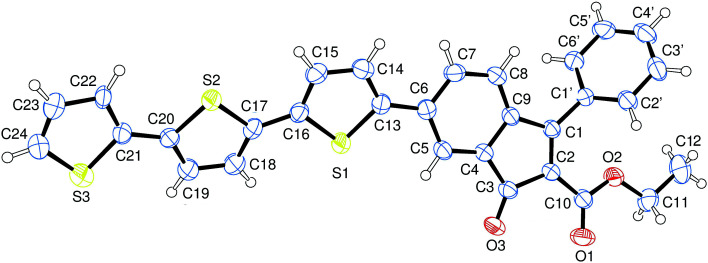
Structure of indenone derivative 2 obtained by crystallography. Ellipsoids enclose 50% probability.

Compound 2 was characterized by crystallography (Fig. 2) and promptly transformed in excellent yields (*ca.* 90%) into corresponding indenol derivative 3 by methylation with trimethylaluminium.

The dehydration of indenol 3 was performed by using diluted solutions in chloroform (or CDCl_3_) containing catalytic amounts of *p*-toluenesulfonic acid (PTSA) and 2,6-di-*tert*-butyl-4-methylphenol (BHT) as an antioxidant in order to obtain solutions of benzofulvene derivative 6-TT-BF3k. After purification by flash chromatography, monomer 6-TT-BF3k was allowed to polymerize spontaneously in the apparent absence of catalysts, upon solvent removal, to give the corresponding poly-6-TT-BF3k. The molecular weight distribution (MWD) of the newly-synthesized poly-6-TT-BF3k was determined by using an absolute multi-angle laser light scattering (MALS) detector on-line to a size exclusion chromatography (SEC) system and found to be in the same range of those measured for similar polybenzofulvene derivatives (see ESI†).

### NMR characterization of the polybenzofulvene derivative bearing terthiophene chromophores 6-TT-BF3k

**Fig. 3:**
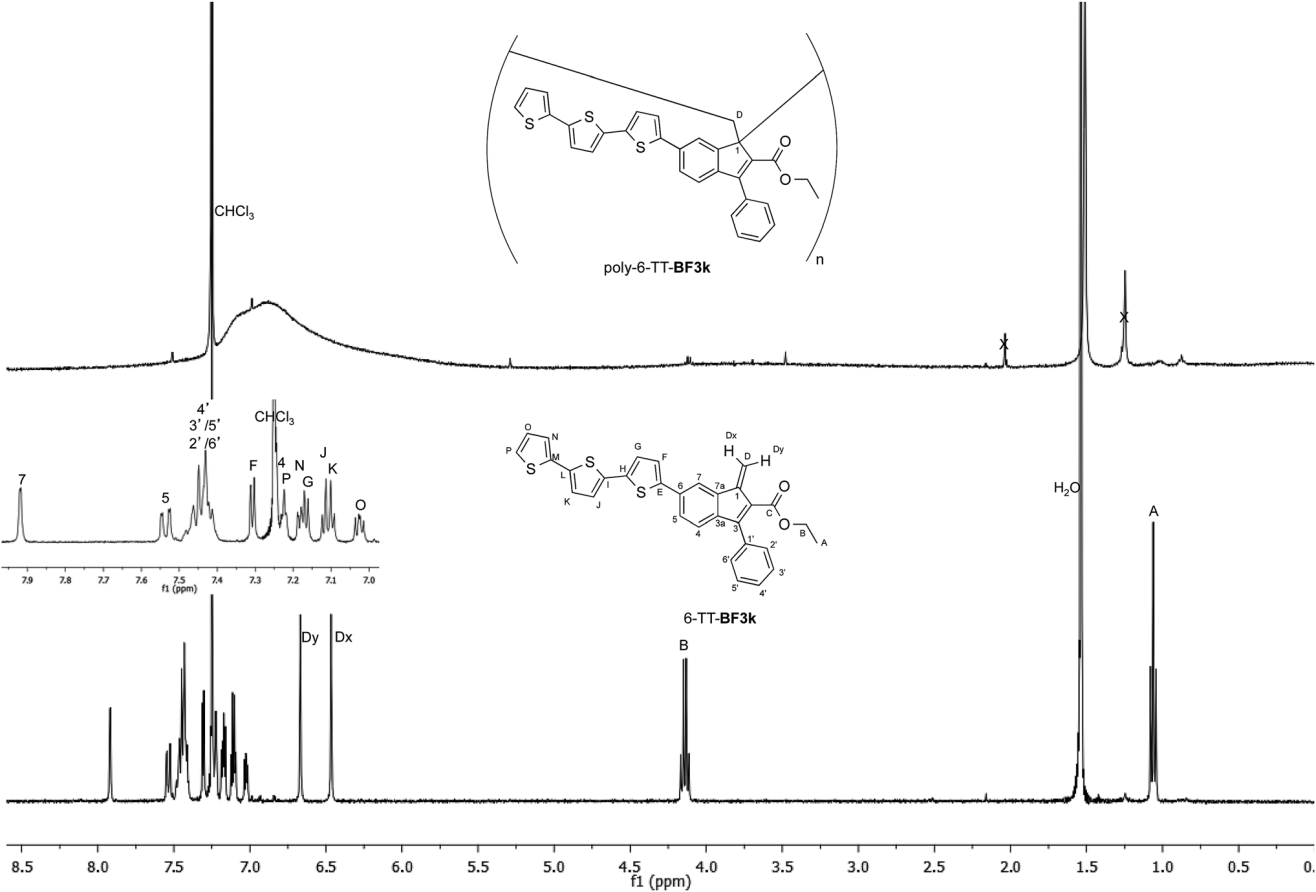
Comparison of the ^1^H NMR spectrum of poly-6-TT-BF3k with that of the corresponding monomer.

**Fig. 4:**
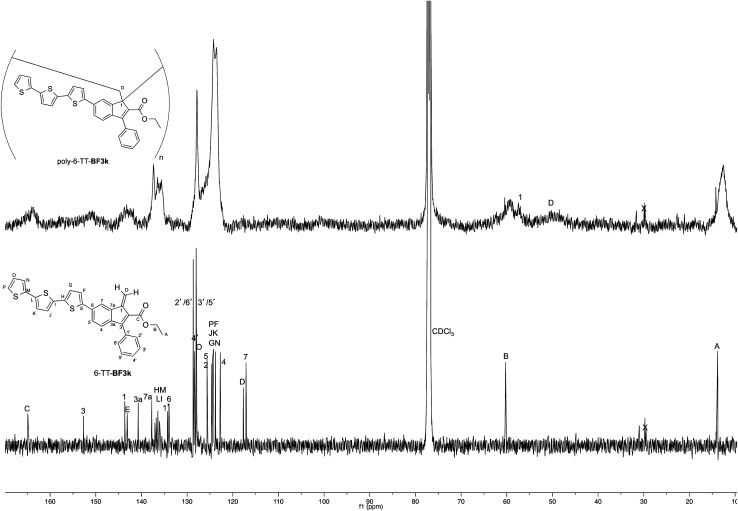
Comparison of the ^13^C NMR spectrum of poly-6-TT-BF3k with that of the corresponding monomer.

The tendency of benzofulvene derivative 6-TT-BF3k towards the spontaneous polymerization typical of benzofulvene monomers was investigated by NMR spectroscopy. In these studies, the ^1^H and ^13^C NMR spectra of monomer 6-TT-BF3k were assigned by means of correlation experiments and compared with those of the corresponding polymer (Fig. 3 and 4).

As expected, the comparison of the proton NMR spectra (Fig. 3) was not very informative because the spectrum of the polymer showed very broad peaks in the correspondence of the sharp ones of the monomer. However, the general up-field of the signals in the polymer spectrum was interpreted as a sign of the presence of strong stacking interactions in the polybenzofulvene macromolecules.

**Fig. 5:**
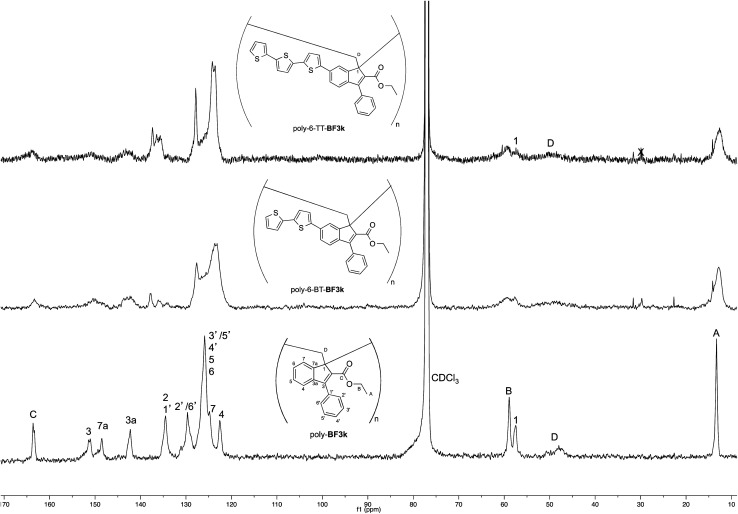
Comparison of the ^13^C NMR spectra of poly-6-TT-BF3k and poly-6-BT-BF3k with that of the parent macromolecule poly-BF3k.

On the other hand, the better resolution obtained in the ^13^C NMR spectrum of the polymer allowed a signal-to-signal comparison to be made between the monomer 6-TT-BF3k and polymer poly-6-TT-BF3k spectra. This NMR characterization allowed the retention of the original vinyl(1,2) polymerization mechanism to be ascertained. In particular, the presence of both the diagnostic peak at around 57 ppm, attributed to the aliphatic quaternary carbon (C-1) of the 1,2-repeating indene unit, and the one at around 49 ppm, attributed to the backbone methylene carbon (C-D), supported the retention of the vinyl polymerization mechanism also in this benzofulvene derivative bearing bulky terthiophene side chains (Fig. 5).^17,22,23^

Furthermore, the comparison of the ^13^C NMR spectra of the polybenzofulvene derivatives bearing the bithiophene or terthiophene chromophores with that of parent poly-BF3k showed that the presence of oligo-thiophene side chains in position 6 of the indene nucleus produced a significant broadening of the signal in the spectra of both poly-6-TT-BF3k and poly-6-BT-BF3k (Fig. 3).

The significant signal broadening was explained by assuming that the presence of the oligo-thiophene side chains in position 6 of the indene nucleus produced a significant increase in the π-stacking among the aromatic moieties of both poly-6-TT-BF3k and poly-6-BT-BF3k that resulted in a decrease of the dynamic features of the monomeric unities.^22^

### Electrochemical characterization of the polybenzofulvene derivative bearing oligo-thiophene chromophores

Benzofulvene monomers 6-BT-BF3k, 6-HBT-BF3k, 4′-BT-6-MO-BF3k, and the corresponding polymers were characterized by cyclic voltammetry, together with poly-6-BT-BF3k-X and the newly synthesized poly-6-TT-BF3k and the most relevant results are summarized in Table 1.

**Table 1:** Redox potentials (in volt *vs.* Ag/AgCl) of benzofulvene and polybenzofulvene derivatives bearing oligothiophene side chains

Compd	*E*°	*E*_CrossLinked_^1^	*E*_CrossLinked_^2^
6-BT-BF3k	+1.15i^a^	+1.30	
6-HBT-BF3k	+1.10i^a^	—	—
4′-BT-6-MO-BF3k	+1.17qr^a^	+0.85	
Poly-6-BT-BF3k-X	+1.35i^a^ broad	—	—
Poly-6-BT-BF3k	+1.13i^a^	+0.67 (+0.88)	+1.10 (+1.10)
Poly-6-TT-BF3k	+1.02	+0.54 (+0.85)	+0.86
Poly-6-HBT-BF3k	+1.10i^a^	—	—
Poly-4′-BT-6-MO-BF3k	+1.04	—	
Poly-4′-HBT-6-MO-BF3k^b^	+1.25i^a^	—	—

a i: chemically irreversible; qr: chemically quasi reversible.

b Poorly soluble.

As indicated in Table 1, the (irreversible) BT processes in the monomers were found in the narrow range from +1.10 to +1.17 V. These values are halfway between those of BT (+1.29 V) and TT (+0.95 V),^27^ indicating the extensive conjugation between the arene and the thiophene rings, and correctly reflect the electron-donor ability of the alkyl chain in 6-HBT-BF3k.

Apart from their close redox potential, the oxidation of these compounds has different consequences, depending on the BT position. In the case of 6-BT-BF3k, the second scan revealed a new, very weak, irreversible process at +1.30 V, which, on the basis of the redox potential value measured for the oxidation of poly-6-BT-BF3k-X (*E*_p_ = +1.35 V, irreversible) may be due to a short oligomer with a similar enchainment, formed upon oxidation. The current was diffusion-controlled and the signal did not grow on repetitive scans, supporting the hypothesis that short, soluble, oligomers were formed, in this case.

As expected, coupling did not occur in the case of 6-HBT-BF3k, which has an engaged α position. The blocking role of the engaged α position with respect to the coupling reaction of the oxidized species was confirmed by the cyclic voltammetry of poly-6-HBT-BF3k, which redox profile is almost superimposable to that of the monomer.

**Scheme 2:**
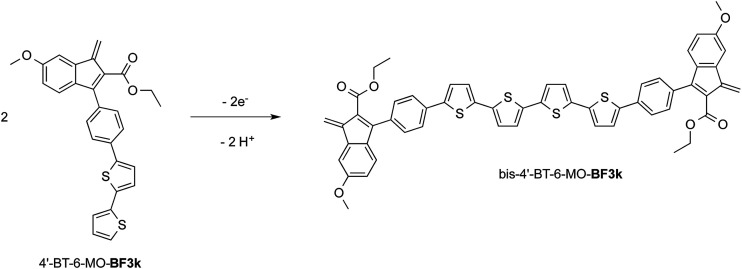
Oxidation of 4′-BT-6-MO-BF3k into the tetrathiophene bridged dimer bis-4′-BT-6-MO-BF3k.

At variance, the oxidation of 4′-BT-6-MO-BF3k was partially chemically reversible but a new, more anodic, oxidation process appeared on the second scan (at +0.85 V) and a very thin film slowly grew, suggesting that a new, low soluble, species was formed, which precipitated on the electrode surface. As it will be discussed successively, this new species could be tentatively identified as the tetrathiophene bridged dimer bis-4′-BT-6-MO-BF3k (Scheme 2).

The redox behavior of the polymers also gave information on the importance of the position occupied by the bithiophene moiety, since 6- or 4′-substituted polymers performed in very different ways. Firstly, the redox potentials of poly-6-BT-BF3k and poly-6-HBT-BF3k were almost unaltered with respect to that of the correspondent monomers, while unexpectedly, the redox potential of poly-4′-BT-6-MO-BF3k was cathodically shifted with respect to the monomer by ∼130 mV. At this stage, we were unable to find any simple explanation for this fact, which, in other cases has been ascribed to an additional stabilization due to the charge delocalization through π-stacking interactions in the polymers.^27^ As matter of fact, the NMR studies suggest that the π-stacking should be more pronounced in 6- than in 4′- substituted polymers.^27^

Then, the cyclic voltammetry of 4′-substituted polymers did not give evidence of any cross-coupling reaction accompanying the removal of one electron, neither in the trivial case of the α-engaged compound poly-4′-HBT-6-MO-BF3k nor in the case of poly-4′-BT-6-MO-BF3k. While, as will be discussed in more detail in the following, oxidation of both the BT and the TT 6-substituted polymers readily gave rise to the formation of a conductive film, due to the inter-chain cross-coupling given by the dimerization of BT or TT moieties.

This latter phenomenon is a very rare occurrence and, apart from the hyperbranched polythiophenes,^27^ to the best of our knowledge, the only other examples have been reported in 1993 by Shirota *et al.* and by Khanna *et al.* who described the chemical and electrochemical synthesis and the characterization of poly-vinyl^27–29^ and polymethacrylate^30^ derivatives, cross-linked by the oxidative coupling of pendant oligothiophenes. Anyway, and at variance with what happens for these benzofulvene polymers, in both those cases the cross-linking reaction occurred during the polymerization process itself.

In the first scan poly-6-BT-BF3k and the newly synthesized poly-6-TT-BF3k underwent a partially reversible oxidation process at +1.13 and +1.02 V, respectively. As in the case of the corresponding monomer 6-BT-BF3k, the redox potential of the BT containing polymer is slightly lower than that of simple BT, but slightly higher than that of simple TT. On the other side, the value found for poly-6-TT-BF3k was rather high for a TT-containing compound and suggested some loss of conjugation, *i.e.* a loss of planarity probably due to the polymer folding.

**Fig. 6:**
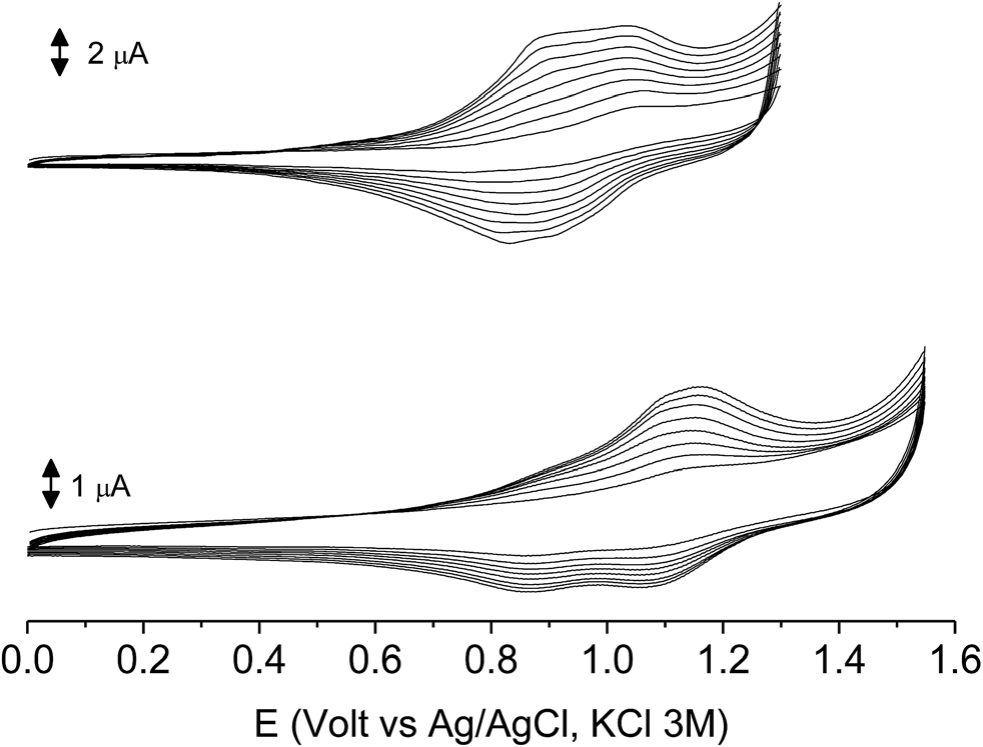
The first 8 cycles in the cyclic voltammetry of poly-6-BT-BF3k (1.8 × 10^−4^ M, bottom) and poly-6-TT-BF3k (4.1 × 10^−4^ M, top) registered on a glassy carbon electrode (GCE) in dichloromethane solutions with [Bu_4_N][PF_6_] (0.1 M) as supporting electrolyte. Scan rate 0.02 V s^−1^.

The subsequent 15–20 scans made a film growing, with two well-defined reversible peaks for poly-6-BT-BF3k (at +0.88 and +1.10 V) and one reversible peak at +0.85 V for poly-6-TT-BF3k (Fig. 6). These peaks were attributed to the formation of tetra- or hexathiophene cross-linked chains of benzofulvene polymers. After ∼40 scans the film became brittle and some flakes detached.

**Fig. 7:**
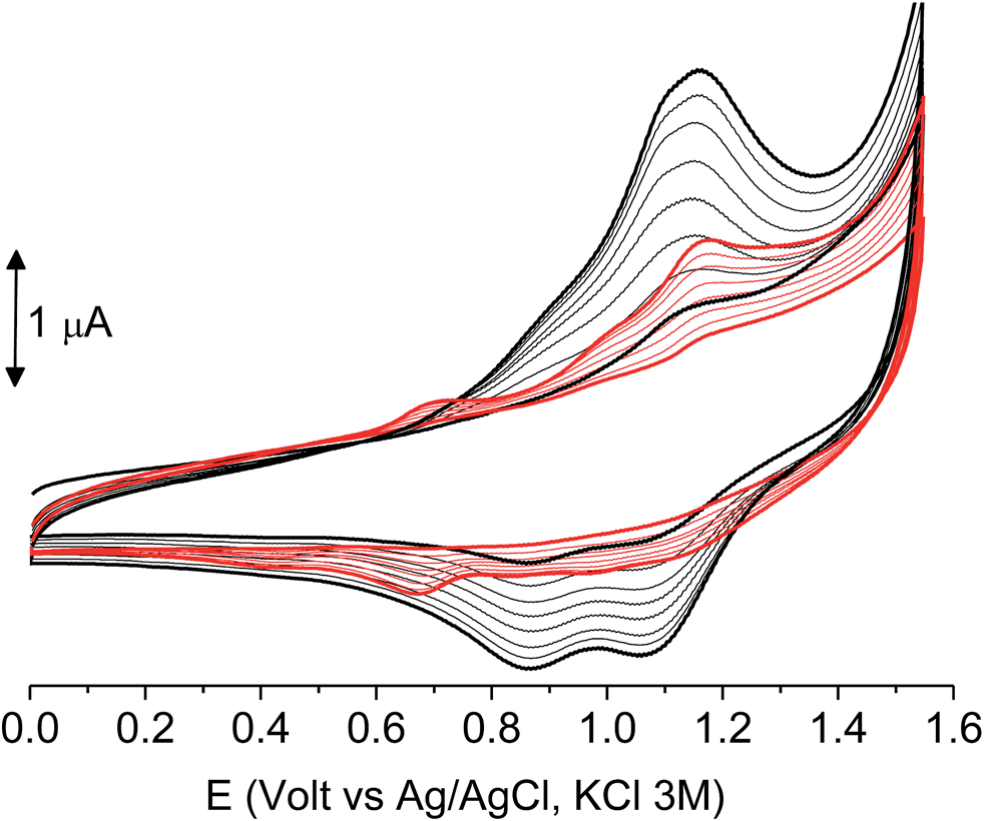
The first 8 cycles in the cyclic voltammetry of diluted (1.0 × 10^−4^ M red) or concentrated (1.8 × 10^−4^ M, black) poly-6-BT-BF3k solutions, registered on a glassy carbon electrode (GCE) in dichloromethane solutions with [Bu_4_N][PF_6_] (0.1 M) as supporting electrolyte. Scan rate 0.02 V s^−1^.

When the experiments were performed in more diluted conditions, a more cathodic peak was also detected at +0.67 V and +0.54 V for poly-6-BT-BF3k and poly-6-TT-BF3k, respectively (Fig. 7). These peaks were never observed when a large indium tin oxide (ITO) glass was used as the working electrode. The presence of multiple peaks is not rare in electropolymerization experiments^4,27^ and may have different origins: they can be due to the presence of longer chains, to the formation of more or less ordered regions with longer or shorter conjugation pathway, or to the kinetics of the inclusion/exclusion process of the counterions accompanying the doping/dedoping process.

In our case, the thiophene polymerization is forcefully blocked, and the chains obtained by cross-coupling possess 4 or 6 units as the fixed length. Thus, the more plausible explanation is that the lower potential peak (observed in diluted conditions) is due to perfect deployment of the coupled chains that is not achievable when a crowded mass of spaghetti quickly precipitates on the electrode surface, as with the large ITO electrode or in a more concentrated solution. In that case, torsion angles may be forced to less convenient positions, the conjugation path is shortened and the redox potential rises.

**Fig. 8:**
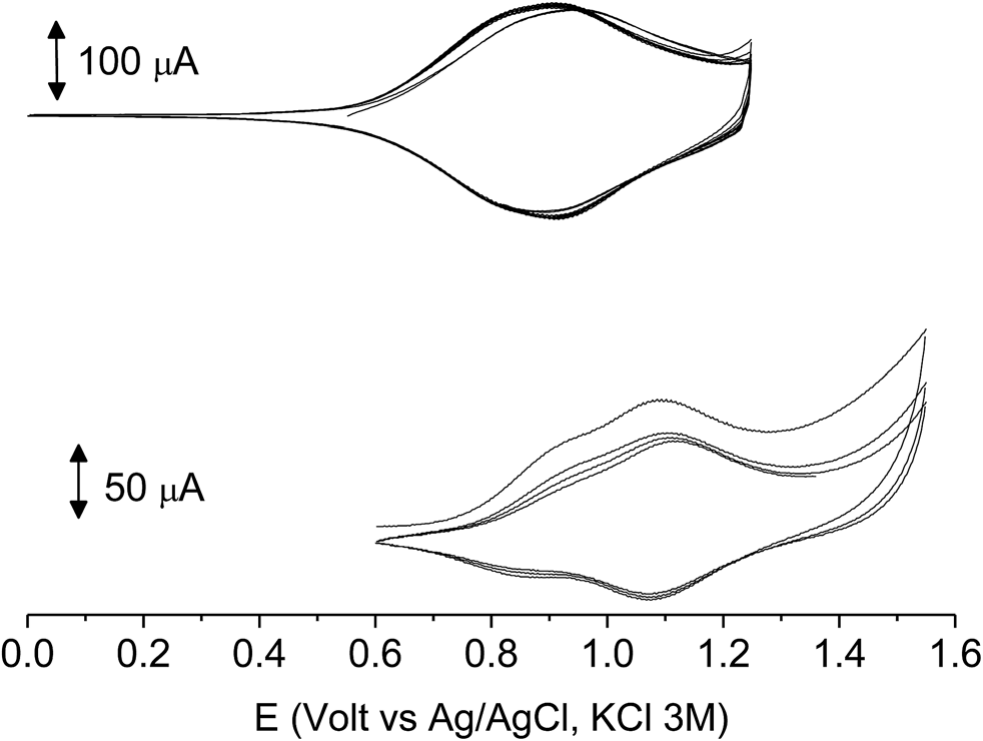
The first 10 cycles in the cyclic voltammetry of poly-6-BT-BF3k (bottom) and poly-6-TT-BF3k (top) films electrodeposited on ITO electrodes recorded in an analyte-free solution. Dichloromethane solutions with [Bu_4_N][PF_6_] (0.1 M) as supporting electrolyte. Scan rate 0.02 V s^−1^.

**Fig. 9:**
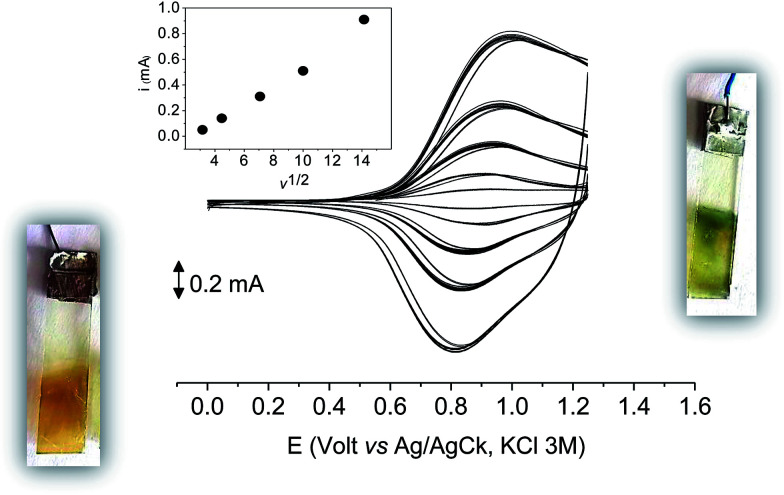
The first 10 cycles in the cyclic voltammetry of poly-6-TT-BF3k film on ITO recorded in an analyte-free solution at various scan rates (from 0.01 to 0.2 V s^−1^) and the current function trend (inset). Dichloromethane solutions with [Bu_4_N][PF_6_] (0.1 M) as supporting electrolyte.

The films deposited on ITO electrodes were tested in an analyte-free electrolyte solution and found to exhibit a diffusion-controlled behavior (inset of Fig. 9), most likely ruled by the PF_6_^−^ inclusion/exclusion during the charge/discharge of the polymer. The peaks remained centered at the initial value without any shift and were broad enough to hide the residual non-coupled pendant thiophene units (Fig. 8), which anyway were certainly still present, as it will be seen discussing the electronic spectra of the films.

With 10 cycles, the surface coverage *Γ* was estimated to be ∼5 × 10^6^ mol cm^−2^ (from eqn (1)) for both poly-6-BT-BF3k and poly-6-TT-BF3k.1
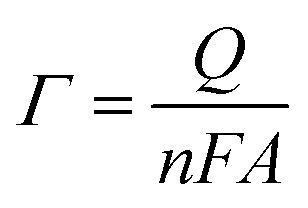


**Fig. 10:**
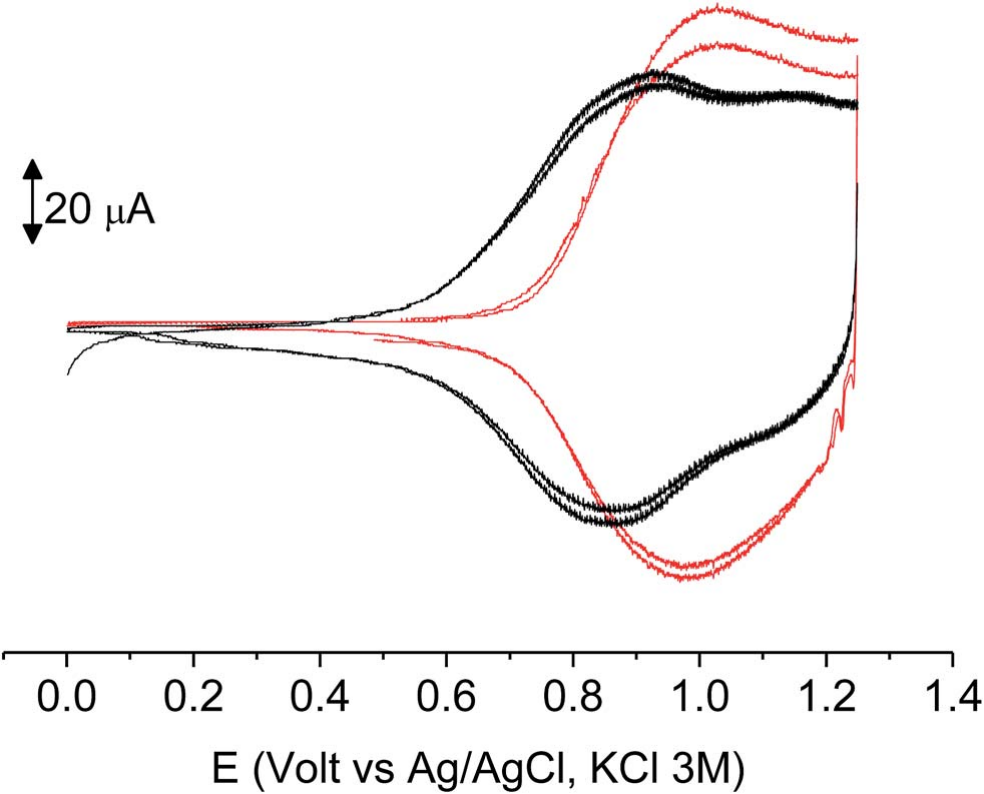
Cyclic voltammetry of freshly prepared poly-6-TT-BF3k film on ITO (black) and after one month of storage in the electrolyte solution (red). Both are recorded in analyte-free solutions. Dichloromethane solutions with [Bu_4_N][PF_6_] (0.1 M) as supporting electrolyte. Scan rate 0.02 V s^−1^.

As it occurs in many thiophene derivatives, the films were electrochromic and switched from yellow-orange (neutral) to green (positively charged) by switching the potential (Fig. 9, see the MOVIE†). The films resisted tenths of cycles, without degradation, but, once dried, the signal was lost and could not be recovered neither by prolonged soaking in the electrolyte solution. It seems reasonable that a similar behavior may be the effect of a collapsed structure of the dried cross-linked polymer, which prevents the ions flux into the network and consequently inhibits the charge/discharge cycle. In fact, when stored one month in the electrolyte solution, a film of poly-6-TT-BF3k still exhibited a cyclic voltammogram with only minor modifications of the signal (Fig. 10).

### Photophysical features of the polybenzofulvene derivative bearing terthiophene chromophores poly-6-TT-BF3k

**Fig. 11:**
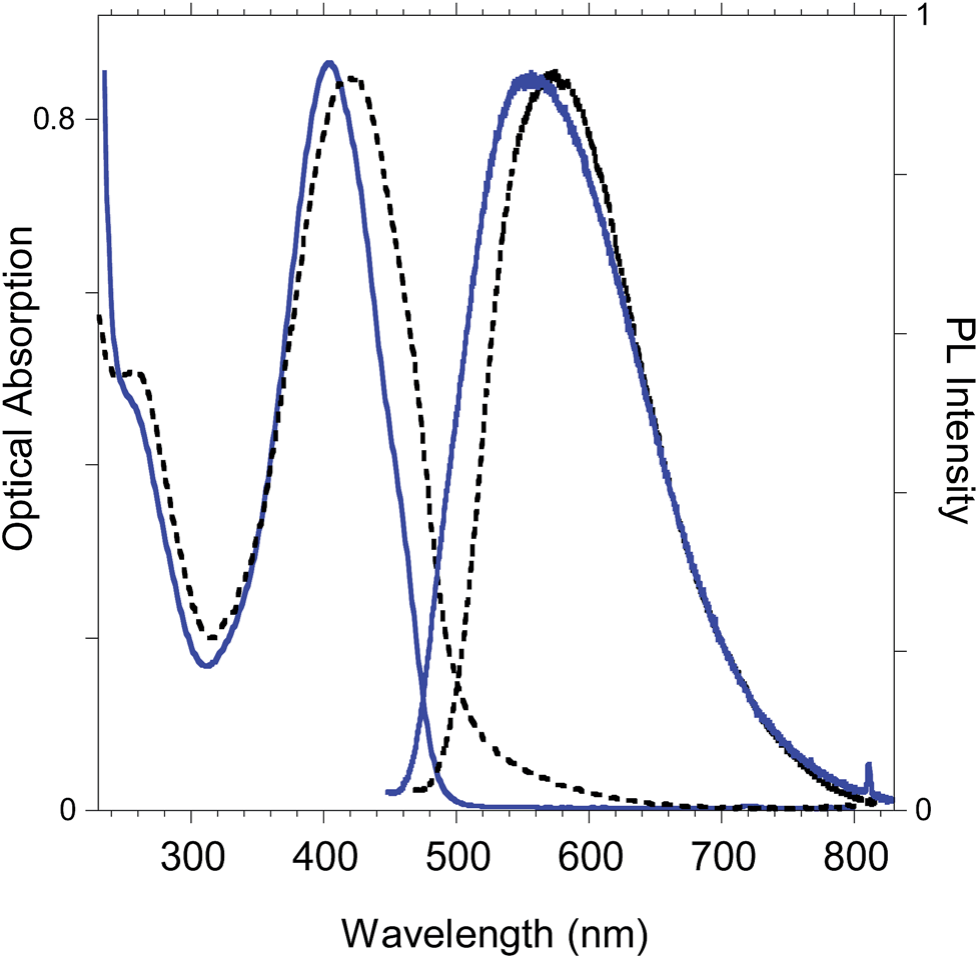
Optical absorption and emission spectrum of poly-6-TT-BF3k in dichloromethane solutions (blue lines) and in the solid state (black lines).

The optoelectronic properties of the newly-synthesized polybenzofulvene derivative poly-6-TT-BF3k (Fig. 11) are summarized in Table 2 for both the solid state and the solution in comparison with those previously reported for the polybenzofulvene derivatives bearing bithiophene moieties (*i.e.* poly-6-BT-BF3k-X, poly-6-BT-BF3k, poly-6-HBT-BF3k, poly-4′-BT-6-MO-BF3k, and poly-4′-HBT-6-MO-BF3k).^22^

**Table 2:** Optoelectronic properties of poly-6-TT-BF3k compared with those shown by poly-6-BT-BF3k-X, poly-6-BT-BF3k, poly-6-HBT-BF3k, poly-4′-BT-6-MO-BF3k, and poly-4′-HBT-6-MO-BF3k^22^

Polymer	Mobility (cm^2^ V^−1^ s^−1^)	Solution			Solid		
		*λ*_ab_ (nm)	*λ*_em_ (nm)	QY (%)	*λ*_ab_ a (nm)	*λ*_em_ b (nm)	QY^b^ (%)
Poly-6-TT-BF3k	5.87 × 10^−5^	405	560	10	420	573	1
Poly-6-BT-BF3k-X		380	575	5	387	640	<0.1
Poly-6-BT-BF3k	7.32 × 10^−5^	380	490	7	386	555	<0.1
Poly-6-HBT-BF3k	5.81 × 10^−5^	386	493	3	394	558	<0.1
Poly-4′-BT-6-MO-BF3k		346	460	12	348	490	9
Poly-4′-HBT-6-MO-BF3k	3.95 × 10^−5^	354	460	14	360	490	13

a Spin coated films.

b Powders.

As shown in Table 2, from the comparison of the optical properties of the whole series of polybenzofulvene derivatives bearing thiophene moieties, it appears evident that when the thiophene moiety is present at position 6, a strong reduction of the photoluminescence (PL) efficiency (*i.e.* quantum yield, QY) is observed, accompanied by a sharp red-shift of the emission. Thiophene oligomers display a high tendency towards aggregation caused quenching (ACQ) processes induced by the strong intermolecular interactions of this class of molecules in the solid state.^31^ The emission behavior of these polymers can be associated to the formation of low emissive excimer states induced by a tight π-stacking of the thiophene based moieties^32^ when the enchainment is at position 6 of the indene group. Differently, the higher emission in the solid state of the corresponding polymers bearing the chromophore in position 4′(poly-4′-BT-6-MO-BF3k and poly-4′-HBT-6-MO-BF3k) is indicative of a weaker chromophore interaction. Therefore, different polymer enchainment and substitution topology of the monomeric units induce different optical properties in the solid state.

Thus, poly-6-BT-BF3k and poly-6-HBT-BF3k bearing the bithiophene chromophore in position 6 display the most efficient side chain packing in the solid state, that is expected to favor the hole mobility of their films.

As previously reported for the polymers with alkyl substituted bithiophene derivatives^22^ the hole mobilities were measured for poly-6-TT-BF3k and poly-6-BT-BF3k (see Table 2) by using as a reference poly(*N*-vinylcarbazole) (PVK), a standard hole-transporting polymer widely used in optoelectronic applications. We used a simple hole-only device structure ITO/PEDOT/MoO_3_/polymer/MoO_3_/Al that gives good ohmic contacts. The mobility of both the two polybenzofulvene derivatives was slightly higher than that of PVK (9.75 × 10^−6^ cm^2^ V^−1^ s^−1^) confirming the good transporting properties of this class of polybenzofulvene derivatives possessing chromophore π-stacking^22^ and nevertheless lower than the best performing PT derivatives in optimized field-effect transistor (FET) devices.^33^

### UV-vis spectroelectrochemistry of the films

**Fig. 12:**
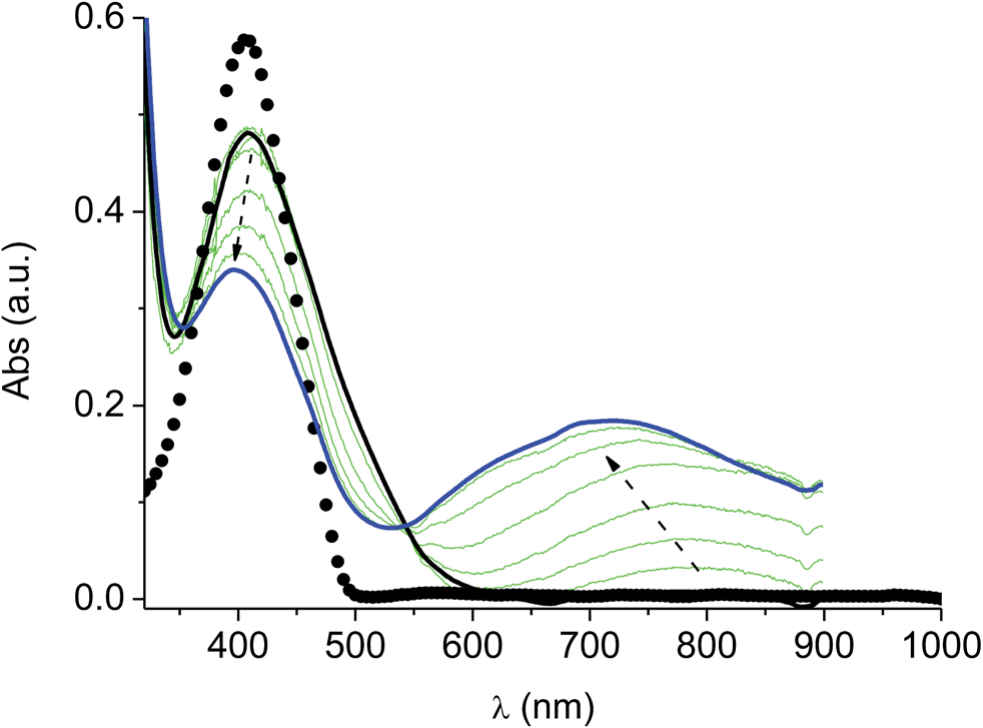
Spectroelectrochemistry of a thin film of poly-6-TT-BF3k deposited on an ITO electrode. The potential was progressively increased from +0.4 V (solid black line) to +1.2 V (solid blue line). The experiment has been recorded in an analyte-free solution. The spectrum of a solution of the pristine poly-6-TT-BF3k is also reported (dotted line).

**Fig. 13:**
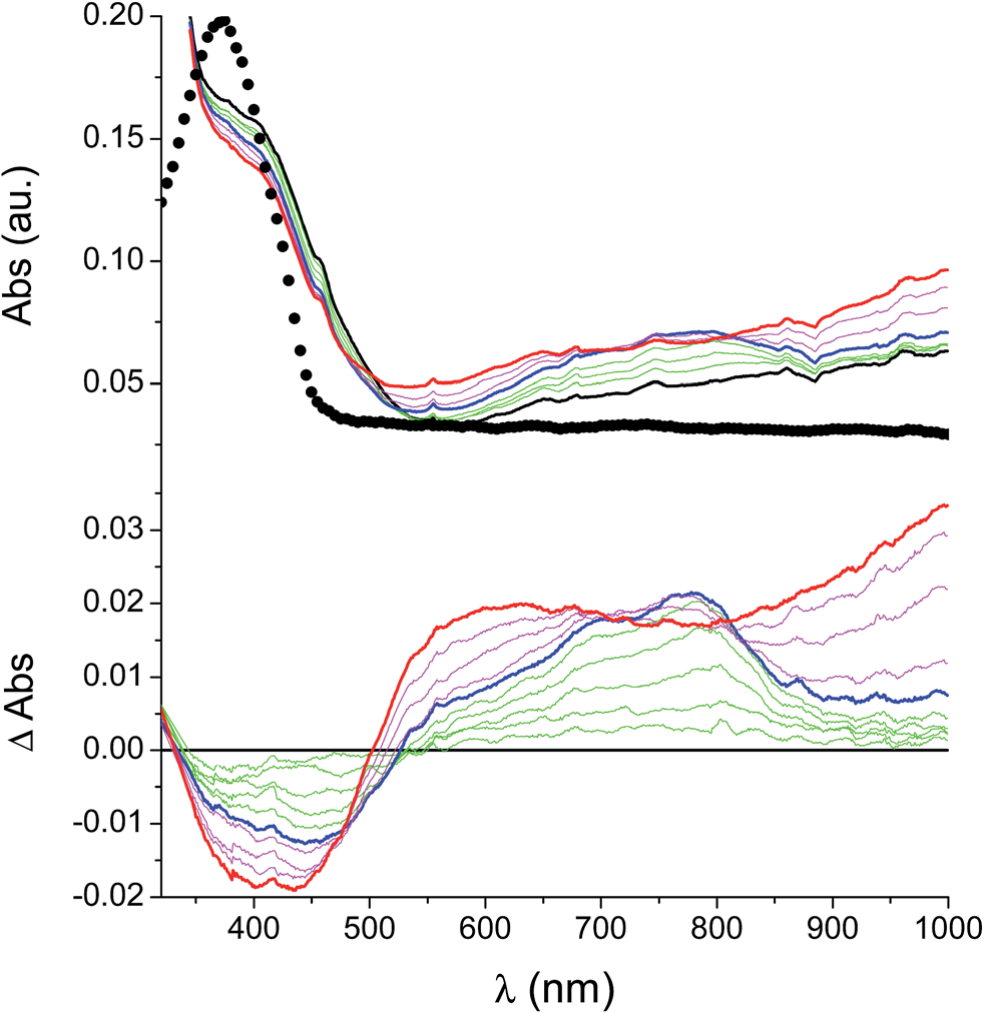
Spectroelectrochemistry (top) and difference spectroelectrochemistry (bottom) of a thin film of poly-6-BT-BF3k deposited on an ITO electrode. The potential has been progressively increased from +0.2 V (black) to +1.1 V (blue) and to +1.3 V (red). The experiment has been recorded in an analyte-free solution. The spectrum of a solution of the pristine poly-6-BT-BF3k is also reported (dotted line).

In the neutral state the film of cross-coupled polymers showed an asymmetric absorption peak centered around 400 nm (*i.e.* poly-6-BT-BF3k: 387 nm; poly-6-TT-BF3k: 410 nm), which resulted to be slightly red-shifted with respect to the spectrum of a solution of the uncoupled polymer (poly-6-BT-BF3k: 370 nm; poly-6-TT-BF3k: 407 nm). The band gap is 2.3 eV or 2.0 eV for poly-6-BT-BF3k and poly-6-TT-BF3k, respectively (onset of the peak at 533 nm and 600 nm), smaller than that of the pristine polymer (poly-6-BT-BF3k: 2.6 eV, onset at 470 nm; poly-6-TT-BF3k: 2.5 eV, ponset at 500 nm). This behavior is characteristic of the enhanced conjugation accomplished both on going from BT to TT containing polymer and with cross-coupling. As the potential was scanned in the positive direction, the intensity of the peak at ∼400 nm decreased, while it was progressively blue-shifted. At the same time a new broad band appeared in the NIR region and constantly increased, shifting to the higher energy region simultaneously (Fig. 12 and 13).

A reasonable interpretation is that the more conjugated (cross-coupled, more ordered, or longer) chains, responsible of the red components of both the neutral (visible) and the cationic (near infrared, NIR) bands, are oxidized at the lower potential, while the less conjugated (not coupled, less ordered, or shorter) chains, responsible of the blue components of both the neutral and the cationic bands, are oxidized at the higher potential. This produces a progressively blue-shifting spectrum. Possibly, when the potential is positive enough to oxidize the less conjugated chains, it may also be positive enough to remove a second electron from the more conjugated chains. This last effect is particularly evident in the difference spectra of poly-6-BT-BF3k, which show the low-energy component of the NIR band (780 nm) increasing in the first steps, followed by the appearance of an higher energy band (ill-defined, at ∼600 nm). The simultaneous appearance of a bipolaronic band at ∼1000 nm, at the expenses of the band at 780 nm is also evident.

**Fig. 14:**
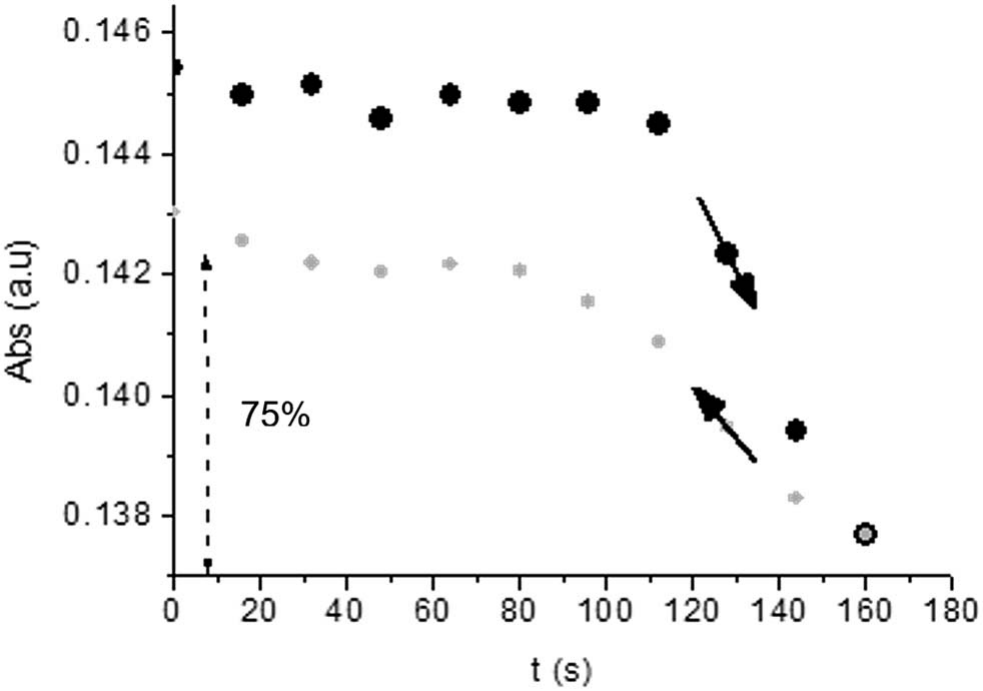
Time-drive absorbance run at 387 nm during the oxidation (+0.5 V to +1.4 V, black dots) and the subsequent reduction (+1.4 V to +0.5 V, grey dots) of a film of poly-6-BT-BF3k. Only 75% of the original absorbance is recovered.

Unlike poly-6-TT-BF3k, the spectra of poly-6-BT-BF3k have an uphill baseline: this is not due to an experimental mishandling and in facts, it has been regularly observed. We have to remind that films were deposited on the ITO surface by repetitive potential scans, then the experiment was stopped at *E* = 0 V to obtain a neutral, undoped film. Ions from the solution should continuously enter and exit into the film channels to maintain electroneutrality. Anyway, the film porosity can be low enough to hamper the ion flux preventing a complete charge/discharge process. This is reasonably the case for the cross-linked poly-6-BT-BF3k, while the longer thiophene chains in poly-6-TT-BF3k are consistent with larger pores. Time-drive absorbance run at 387 nm, clearly indicates that in a two-scan experiment (from +0.5 V to +1.4 V and back) with scan rate 5 mV s^−1^ poly-6-BT-BF3k can release only 75% of its charge (Fig. 14).

**Fig. 15:**
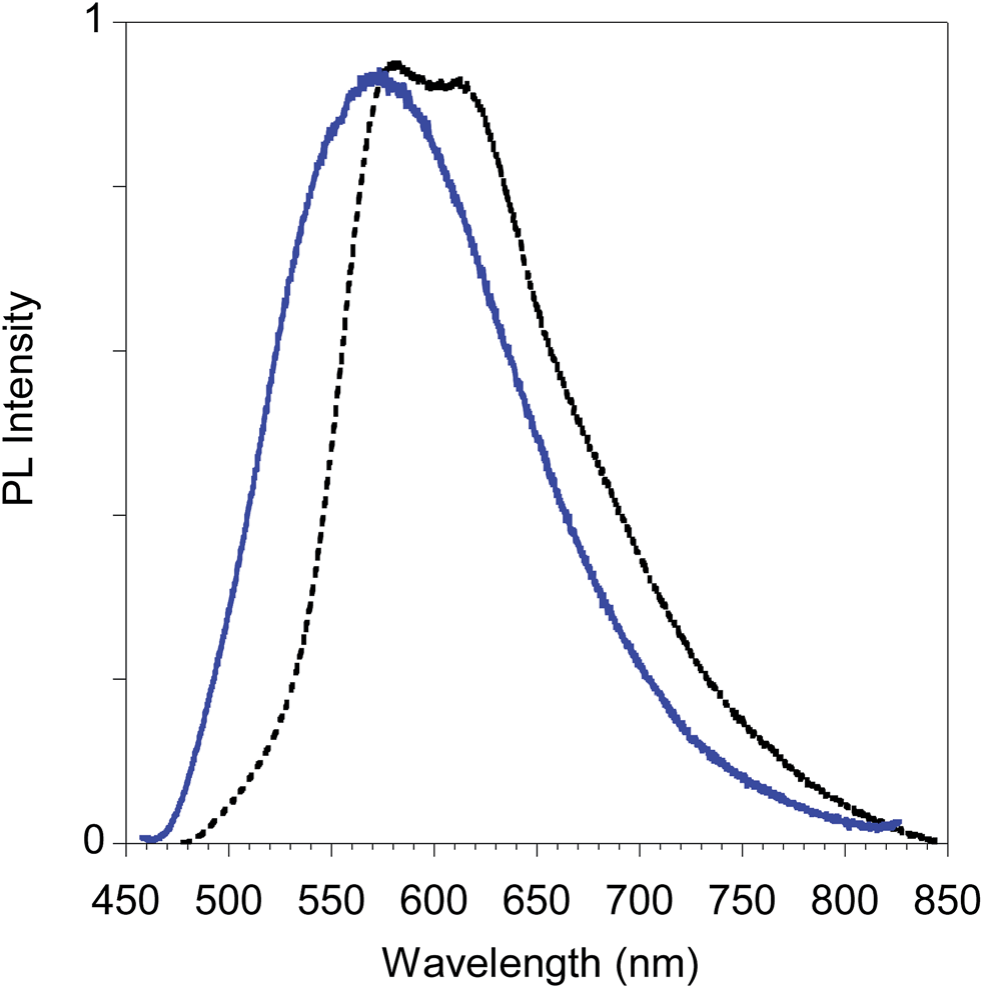
PL spectra of poly-6-TT-BF3k film obtained by electrochemical deposition on the ITO electrode (black dotted line) and by casting of a dichloromethane solution (blue solid line).

Finally, in Fig. 15 we report the PL spectrum of the electrodeposited thin film of poly-6-TT-BF3k compared with a film obtained by casting a dichloromethane solution.

The red-shift of the spectrum of the electrodeposited film, that shows a band peaked at about 615 nm, is consistent with the presence of more conjugated moieties in the electrodeposited film, such as cross-coupled, more ordered or longer chains.^31,34^

## Conclusion

The members of the subfamily of polybenzofulvene derivatives bearing bithiophene and terthiophene chromophores were studied in a systematic electrochemical characterization by cyclic voltammetry in comparison with the corresponding monomers. Very interestingly, the presence of unsubstituted terminal thiophene moieties allowed poly-6-BT-BF3k and poly-6-TT-BF3k to be cross-linked by electrochemical procedures into new materials showing potentially unprecedented properties. Thus, conductive films of polybenzofulvene derivatives were obtained by electrodeposition from solutions of poly-6-BT-BF3k or poly-6-TT-BF3k onto electrode surfaces through the formation of covalent inter-chain cross-linking due to oxidative coupling of the BT or TT side chains, respectively. These films showed electrochromic features and switched from yellow-orange (neutral) to green (positively charged) by switching the potential. Moreover, the films were stable to tenths of cycles, without degradation in the wet state for up to one month in the electrolyte solution, but they lost some of their properties once dried. Finally, the film obtained by electrodeposition from a solution of poly-6-TT-BF3k showed in the neutral state a significantly red-shifted PL emission (∼40 nm) with respect to that of the corresponding film obtained by casting procedures. This occurrence was consistent with the presence of more conjugated moieties in the electrodeposited film. These results pave the way for the preparation of new cross-linked hybrid materials based on π-stacked polybenzofulvene backbones bearing oligothiophene side chains that, by virtue of their innovative architecture and easy preparation, could find a broad range of application in optoelectronics and bioelectronics.

## Experimental section

### Synthesis

The details of the preparation of benzofulvene derivatives and their spontaneous polymerization are described in (ESI†). NMR spectra were recorded with either a Bruker DRX-400 AVANCE or Bruker DRX-500 AVANCE spectrometer in the indicated solvents (TMS as internal standard): the values of the chemical shifts are expressed in ppm and the coupling constants (*J*) in Hz. An Agilent 1100 LC/MSD operating with an electrospray source was used in mass spectrometry experiments.

### X-ray crystallography

Single crystals of indenone 2 were submitted to X-ray data collection on an Oxford-Diffraction Xcalibur Sapphire 3 diffractometer with a graphite monochromated Mo-Kα radiation (*λ* = 0.71073 Å) at 293 K. The structures were solved by direct methods implemented in SHELXS-97 program.^35^ The refinements were carried out by full-matrix anisotropic least-squares on F^2^ for all reflections for non-H atoms by means of the SHELXL-97 program.^36^ Crystallographic data (excluding structure factors) for the structure in this paper have been deposited with the Cambridge Crystallographic Data Centre as supplementary publication no. CCDC 1560193. Copies of the data can be obtained, free of charge, on application to CCDC, 12 Union Road, Cambridge CB2 1EZ, UK; (fax: +44(0) 1223 336 033; or e-mail: deposit@ccdc.cam.ac.uk).

### Electrochemical studies

In all the experiments N_2_-saturated solutions of the compound under study were used with [Bu_4_N][PF_6_] (0.1 M) as supporting electrolyte (Fluka, electrochemical grade) and freshly distilled dichloromethane. Cyclic voltammetry was performed in a three-electrode cell containing either a glassy carbon or an ITO electrode (Sigma-Aldrich coated glass slide, 15 Ω sq^−1^, 2 cm^2^) working electrode, a platinum counter electrode, and an AgCl/Ag (KCl 3 M) reference electrode. A BAS 100 W electrochemical analyzer was used as polarizing unit. All the potential values are referred to the AgCl/Ag (KCl 3 M). Typical analyte concentration was 10^−3^ to 10^−4^ M in terms of repeating units.

### Optical measurements

UV-vis absorption spectra were obtained with a Perkin Elmer Lambda 900 spectrometer. PL spectra were obtained with a SPEX 270 M monochromator equipped with a N_2_ cooled CCD by exciting with a monochromated 450 W Xe lamp and corrected for the instrument response. PL QY of solutions were obtained by using quinine sulfate as reference. PL QY of solid powders were measured with a home-made integrating sphere according to the procedure reported elsewhere.^37^

### Devices preparation and characterization

Glasses covered with indium tin oxide (ITO, 15 Ω sq^−1^) were cleaned ultrasonically in distilled water, acetone, and isopropanol. On the substrates, a water solution of poly-(3,4-ethylenedioxythiophene)-poly-(styrenesulfonic acid) (PEDOT:PSS, Clevios P VP AI 4083, H. C. Starck) was spincoated through a nylon filter (pore size 0.45 μm), to obtain a layer of 50 nm thickness. The substrates were annealed for 10 min at 100 °C in nitrogen atmosphere. On the substrates 10 nm of MoO_3_ were evaporated at a pressure of 10^−7^ mbar, creating ohmic contact for hole injection. In a nitrogen filled glovebox, organic films were spincoated from a chloroform solution with concentration 15 mg mL^−1^. The obtained layers showed thicknesses values between 200 nm and 300 nm as evaluated by using a Dektak XT (Bruker) profilometer. Top electrodes consisting of 7 nm of MoO_3_ and 80 nm of Al were thermally evaporated in vacuum. The current density–voltage curves were obtained with a Keithley 2602 source meter in a nitrogen atmosphere.

## Conflicts of interest

The authors declare no competing financial interest.

## Supplementary Material

RA-008-C7RA13242E-s001

RA-008-C7RA13242E-s002

RA-008-C7RA13242E-s003
