# Supplementary material for: Electrochemical and optoelectronic properties of terthiophene- and bithiophene-based polybenzofulvene derivatives
Source: RSC Adv. 2018 Mar 19;8(20):10836–47. doi: 10.1039/c7ra13242e (PMC9078966; doi:10.1039/c7ra13242e)
Supplement: RA-008-C7RA13242E-s002 [file RA-008-C7RA13242E-s002.pdf]

Electronic Supplementary Material (ESI) for RSC Advances.
This journal is © The Royal Society of Chemistry 2018

Electronic Supplementary Information

Electrochemical and Optoelectronic Properties of Terthiophene- and Bithiophene-Based Polybenzofulvene Derivatives

Fabrizia Fabrizi de Biani,[*,a] *Annalisa Reale,*[a] *Vincenzo Razzano,*[a] *Marco Paolino,*[a] *Germano Giuliani,*[a] *Alessandro Donati,*[a] *Gianluca Giorgi,*[a] *Wojciech Mróz,*[b] *Daniele Piovani,*[b] *Chiara Botta,*[b] *Andrea Cappelli.*[*,a]

[a]Dipartimento di Biotecnologie, Chimica e Farmacia and European Research Centre for Drug Discovery and Development, Università degli Studi di Siena, Via Aldo Moro 2, 53100 Siena, Italy

[b]Istituto per lo Studio delle Macromolecole (CNR), Via A. Corti 12, 20133 Milano, Italy

Content:

Macromolecular characterization of poly-6-TT-**BF3k** (page 2)

J/V characteristics of the polymers (pages 3-5)

Cyclic voltammograms of the compounds (pages 6 and 7)

Experimental details for the preparation and the characterization of the newly-synthesized compounds (pages 8-14)

Macromolecular characterization of poly-6-TT-BF3k.

An absolute multi-angle laser light scattering (MALS) detector on-line to a size exclusion chromatography (SEC) system (THF as the mobile phase) was employed in the characterization of molecular weight distribution (MWD), size of macromolecules (radius of gyration Rg), and conformation of the polybenzofulvene derivative. The SEC-MALS system and the corresponding experimental conditions were identical to those used in our previous studies[1-3] and are not reported in detail here. The dn/dc value of the polymer (0.250 mL/g) was measured off-line in THF at 35 °C using a Chromatix KMX-16 differential refractometer. Thus, the polybenzofulvene derivative obtained by spontaneous polymerization of 6-TT-**BF3k** featured particularly high average molecular weight values and broad dispersity (D = M_w/M_n) similar to the ones shown by parent polybenzofulvenes (Table 1).[1]

Table ESI-1. Macromolecular features of poly-6-TT-**BF3k** obtained by spontaneous polymerization compared with those shown by parent polymers.

polymer	M_p (kg/mol)	M_w [a] (kg/mol)	M_w/M_n [b]	R_g [c] (nm)	K [d] (nm)	α [d]
poly-6-TT-**BF3k**	1,400	850	4.4	26.4	6.89·E-03	0.57
poly-6-BT-**BF3k**	2,970	2,000	3.5	43.7	1.38·E-02	0.53
poly-6-HBT-**BF3k**	4,700	4,600	2.5	72.7	2.16·E-02	0.51
poly-4'-BT-6-MO-**BF3k**	3,900	2,800	5.2	55.3	2.32·E-02	0.50
poly-4'-HBT-6-MO-**BF3k**	3,800	3,400	2.9	66.2	2.53·E-02	0.51
poly-6-MO-**BF3k**	310	350	4.3	19.4	5.75·E-03	0.61
poly-**BF3k**	1,900	1,500	3.4	49.9	6.63·E-03	0.60

[a] M_w: weight-average molecular weight; [b] M_w/M_n: dispersity where M_n denotes the numeric-average molecular weight; [c] R_g: radius of gyration i.e. dimension of the macromolecules; [d] K, α: intercept and slope of conformation plot.

Mobilities measured for a set of devices and their averages (bold values)

**PVK**	poly-6-BT-**BF3k**	poly-6-TT-**BF3k**
9,50E-06	7,70E-05	5,20E-05
1,00E-05	5,80E-05	6,90E-05
	7,20E-05	6,00E-05
	7,70E-05	5,80E-05
	7,80E-05	5,60E-05
	7,70E-05	5,70E-05
**9,75E-06 cm²/Vs**	**7,32E-05 cm²/Vs**	**5,87E-05 cm²/Vs**

PVK:

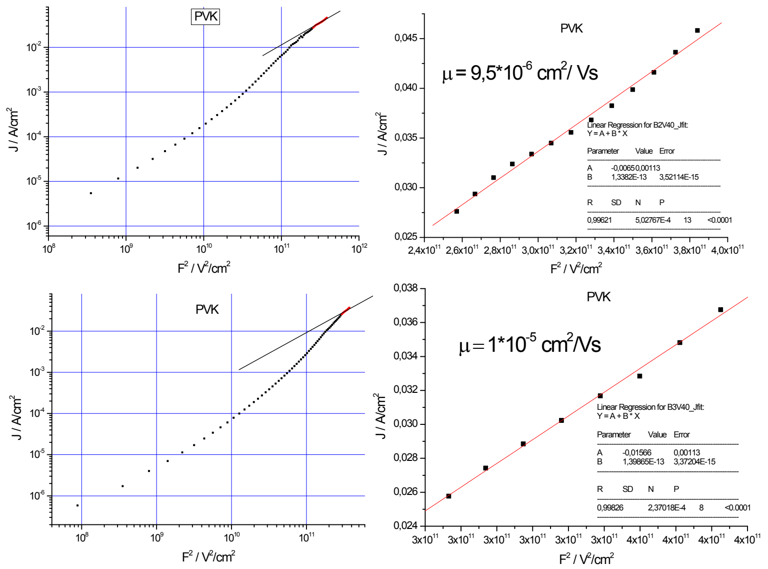

poly-6-BT-**BF3k**:

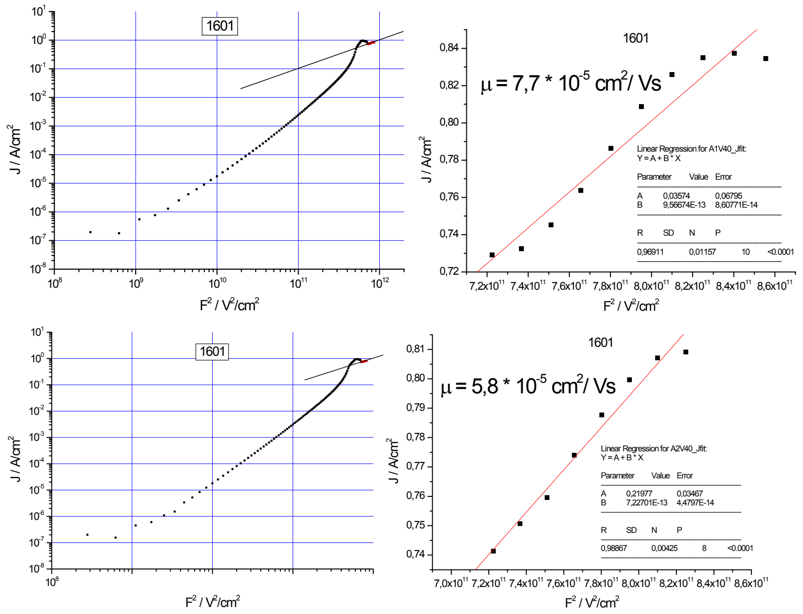

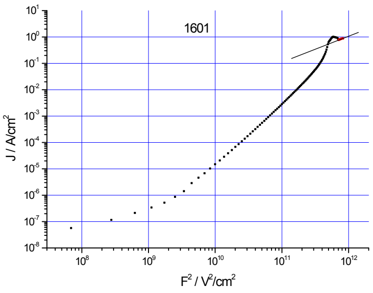

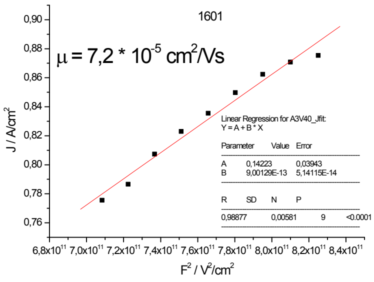

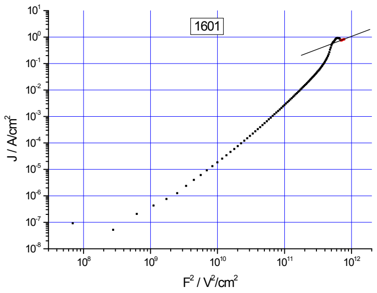

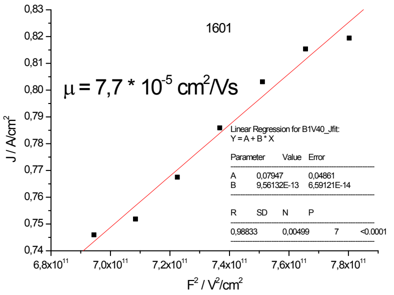

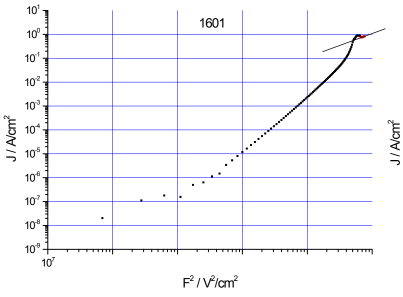

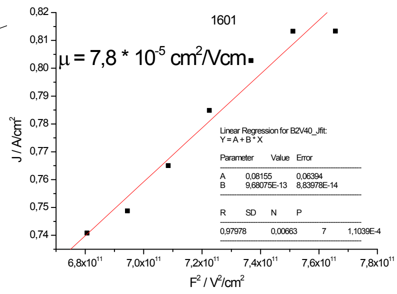

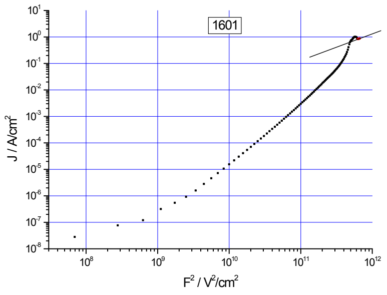

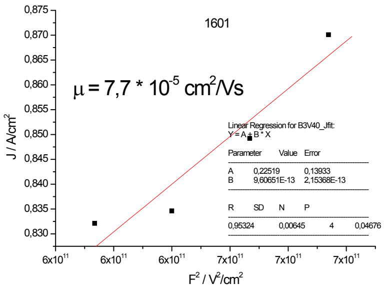

poly-6-TT-**BF3k**:

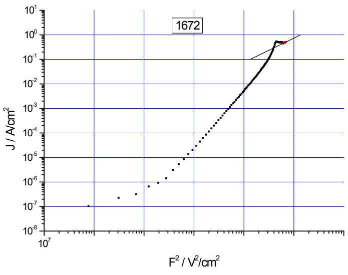

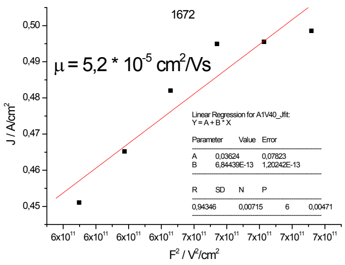

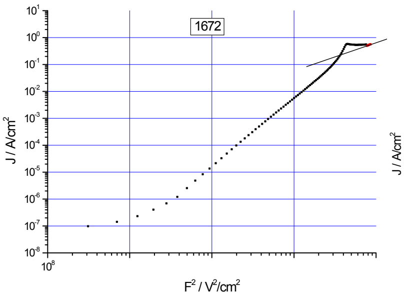

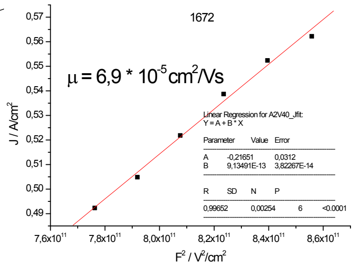

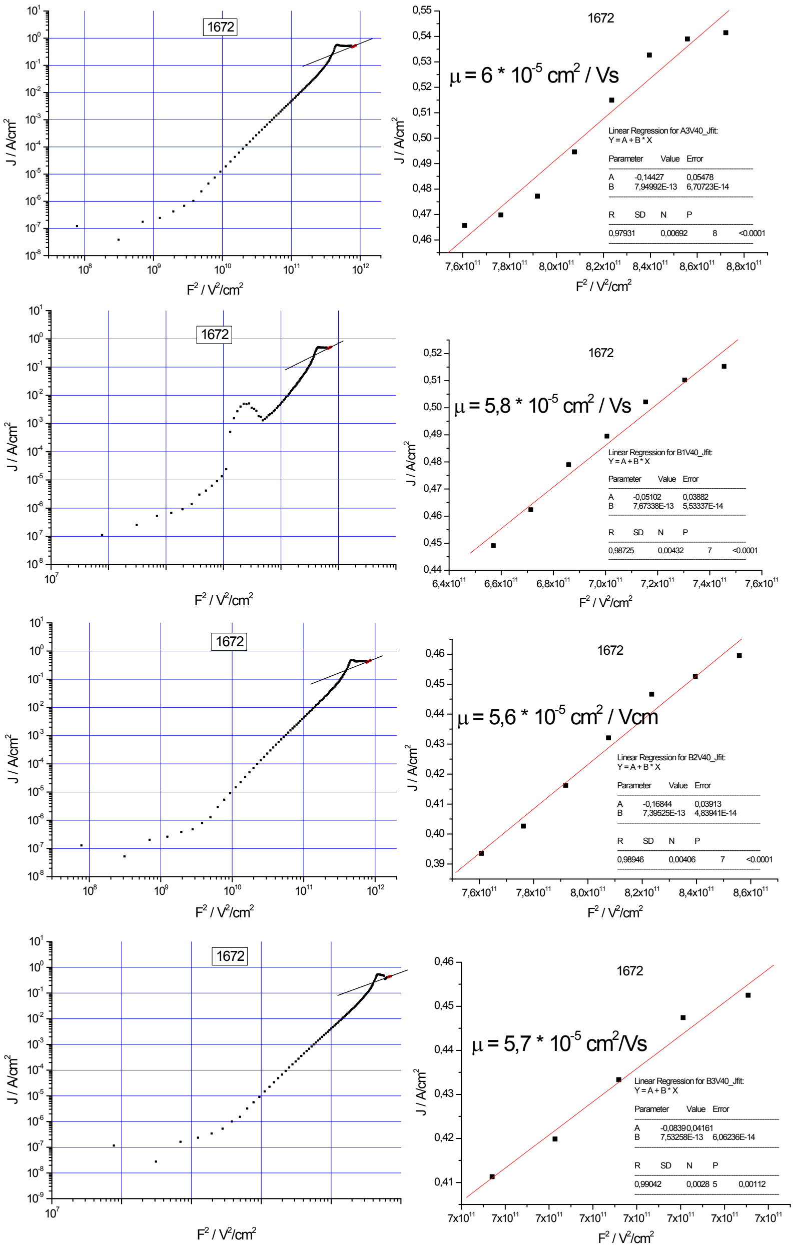

Figure ESI-1. J/V characteristics of poly-6-TT-**BF3k** (bottom), poly-6-BT-**BF3k** (middle) and PVK (top).

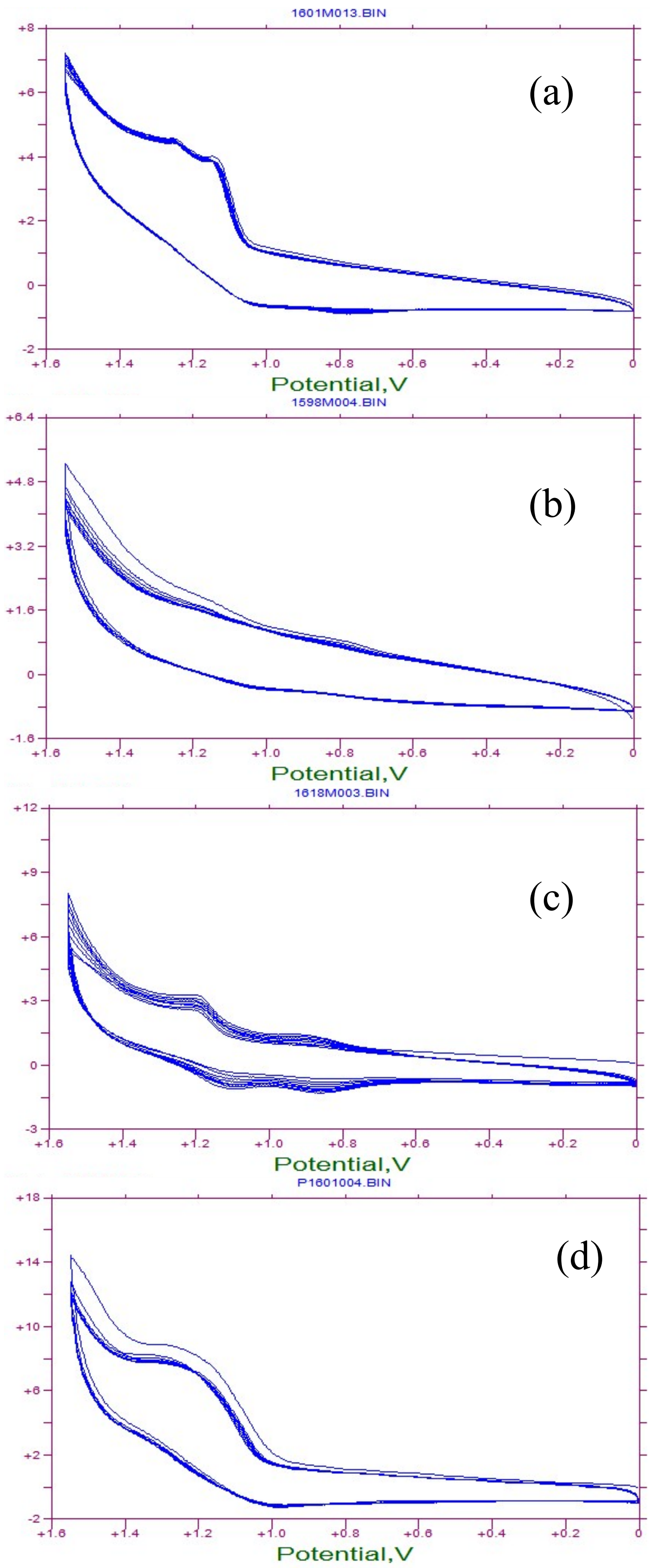
1601M013.BIN
(a)
+8
+6
+4
+2
0
-2
+1.6
+1.4
+1.2
+1.0
+0.8
+0.6
+0.4
+0.2
0
Potential,V
1598M004.BIN
(b)
+6.4
+4.8
+3.2
+1.6
0
-1.6
+1.6
+1.4
+1.2
+1.0
+0.8
+0.6
+0.4
+0.2
0
Potential,V
1618M003.BIN
(c)
+12
+9
+6
+3
0
-3
+1.6
+1.4
+1.2
+1.0
+0.8
+0.6
+0.4
+0.2
0
Potential,V
P1601004.BIN
(d)
+18
+14
+10
+6
+2
-2
+1.6
+1.4
+1.2
+1.0
+0.8
+0.6
+0.4
+0.2
0
Potential,V

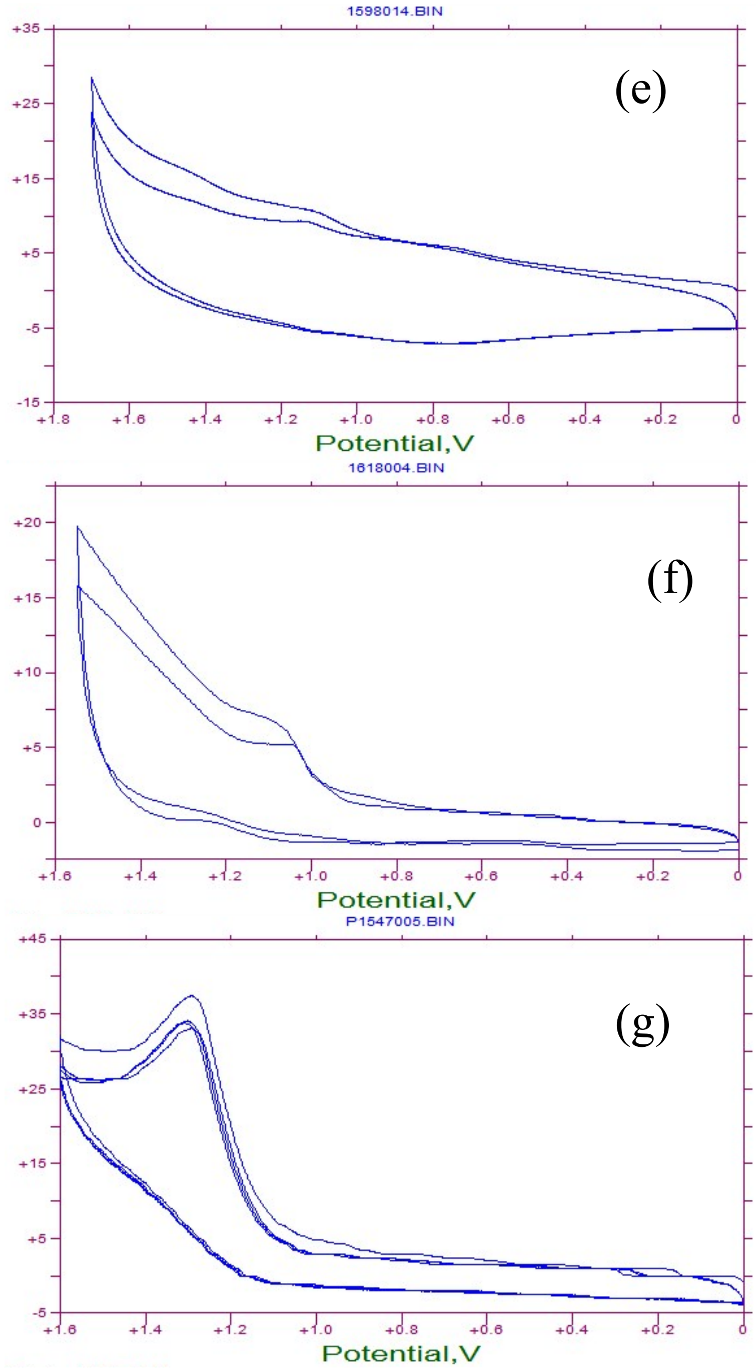

Figure ESI-2. Cyclic voltammetry of 6-BT-**BF3k** (a)**,** 6-HBT-**BF3k** (b), 4'-BT-6-MO-**BF3k** (c), poly-6-BT-**BF3k-X** (d), poly-6-HBT-**BF3k** (e), poly-4'-BT-6-MO-**BF3k** (f) and poly-4'-HBT-6-MO-**BF3k** (g) registered on a glassy carbon electrode (GCE) in dichloromethane solutions with $[Bu_4N][PF_6]$ (0.1 M) as supporting electrolyte. Scan rate 0.2 V s^{-1}.

Experimental details

Synthesis. Melting points were determined in open capillaries in a Gallenkamp apparatus and are uncorrected. Merck silica gel 60 (230-400 mesh) was used for column chromatography. Merck TLC plates, silica gel 60 F_{254} were used for TLC. NMR spectra were recorded with a Bruker DRX-400 AVANCE or a Bruker DRX-500 AVANCE spectrometer in the indicated solvents (TMS as internal standard): the values of the chemical shifts are expressed in ppm and the coupling constants (*J*) in Hz. An Agilent 1100 LC/MSD operating with an electrospray source was used in mass spectrometry experiments.

Ethyl 6-(2,2':5',2"-terthiophen-5-yl)-1-oxo-3-phenyl-1*H*-indene-2-carboxylate (2).

To a solution of 2,2':5',2"-terthiophene-5-boronic acid pinacol ester (176 mg, 0.470 mmol), were added potassium phosphate tribasic (299 mg, 1.41 mmol) and 2,6-di-*tert*-butyl-4-methylphenol (BHT, 52 mg, 0.236 mmol) in 1,4-dioxane (8.0 mL) and anhydrous ethanol (1.0 mL). After stirring the reaction mixture at room temperature for 10 min, bis(triphenylphosphine)palladium(II) dichloride (65 mg, 0.093 mmol), triphenylphosphine (12 mg, 0.047 mmol) and compound **1** (ref 4) (200 mg, 0.469 mmol) were added in sequence. The reaction mixture was heated to reflux for 3 h under a nitrogen atmosphere and then concentrated under reduced pressure. The residue was partitioned between dichloromethane and water. The organic layer was dried over sodium sulfate and concentrated under reduced pressure. Purification of the residue by flash chromatography with dichloromethane as the eluent gave compound **2**, which was further purified by recrystallization from dichloromethane-ethyl acetate by slow evaporation to obtain dark violet prisms (148 mg, yield 60%, mp 193-195 °C). ^{1}H NMR (400 MHz, $CDCl_3$): 1.17 (t, *J* = 7.1, 3H), 4.21 (q, *J* = 7.1, 2H), 7.01-7.05 (m, 1H), 7.10 (d, *J* = 3.6, 1H), 7.13 (d, *J* = 3.8, 1H), 7.15-7.21 (m, 3H), 7.23 (d, *J* = 5.1, 1H), 7.34 (d, *J* = 3.8, 1H), 7.53 (s, 5H), 7.57-7.61 (m, 1H), 7.85 (d, *J* = 1.5, 1H). MS (ESI): *m/z* 547 ($M + Na^+$).

Ethyl 6-(2,2':5',2''-terthiophen-5-yl)-1-hydroxy-1-methyl-3-phenyl-1*H*-indene-2-carboxylate (3).

To a solution of **2** (150 mg, 0.286 mmol) in dichloromethane (15 mL) was added a 2M solution of trimethylaluminium in toluene (0.57 mL, 1.14 mmol). After stirring at room temperature for 30 min under a nitrogen atmosphere, the reaction mixture was diluted with ethyl acetate (15 mL) and the $Al(CH_3)_3$ excess was cautiously destroyed with a 1M sodium hydroxide solution. The resulting mixture was partitioned between ethyl acetate and water, and the organic layer was dried over sodium sulfate and concentrated under reduced pressure. Purification of the residue by flash chromatography with petroleum ether-dichloromethane-ethyl acetate (7:2:1) as the eluent afforded indenol derivative as a green glassy solid **3** (140 mg, yield 90.5%). An analytical sample was obtained by recrystallization from dichloromethane-ethyl acetate by slow evaporation (mp 210-211 °C). ^{1}H NMR (500 MHz, $CDCl_3$): 1.06 (t, J = 7.1, 3H), 1.83 (s, 3H), 3.73 (br s, 1H), 4.08-4.20 (m, 2H), 7.02 (dd, J = 5.0, 3.6, 1H), 7.09 (d, J = 3.8, 1H), 7.11 (d, J = 3.8, 1H), 7.14-7.17 (m, 2H), 7.17-7.20 (m, 1H), 7.22 (dd, J = 5.1, 0.9, 1H), 7.32 (d, J = 3.8, 1H), 7.39-7.40 (m, 2H), 7.43-7.47 (m, 3H), 7.52 (dd, J = 7.9, 1.7, 1H), 7.81 (d, J = 1.4, 1H). ^{13}C NMR (125 MHz, $CDCl_3$): 13.7, 25.9, 60.4, 81.8, 119.5, 123.8, 124.1, 124.4, 124.6, 125.9, 127.9, 128.5, 128.6, 133.4, 135.1, 135.8, 136.0, 136.4, 137.1, 140.3, 142.7, 150.6, 151.6, 165.1. MS (ESI): m/z 563 (M + Na^+).

Ethyl 6-(2,2':5',2''-terthiophen-5-yl)-1-methylene-3-phenyl-1*H*-indene-2-carboxylate (6-TT-BF3k).

A mixture of indenol **3** (5.0 mg, 0.00925 mmol) in $CDCl_3$ (10 mL) containing p-toluenesulfonic acid monohydrate (1.5 mg, 0.00789 mmol) and 2,6-di-*tert*-butyl-4-methylphenol (2.0 mg, 0.00908 mmol) was heated to reflux for 30 min. The reaction mixture was cooled to room temperature and washed in sequence with a saturated solution of sodium bicarbonate and with water. The organic layer was dried over sodium sulfate, concentrated to a volume of ca. 1 mL and purified by flash chromatography with $CDCl_3$ to obtain a solution of pure monomer 6-TT-**BF3k**, which was used in

the NMR studies. ^{1}H NMR (400 MHz, $CDCl_3$): 1.06 (t, *J* = 7.1, 3H), 4.14 (q, *J* = 7.1, 2H), 6.47 (s, 1H), 6.67 (s, 1H), 7.03 (dd, *J* = 5.1, 3.7, 1H), 7.09-7.12 (m, 2H), 7.16-7.19 (m, 2H), 7.22-7.24 (m, 2H), 7.31 (d, *J* = 3.8, 1H), 7.40-7.49 (m, 5H), 7.54 (dd, *J* = 8.0, 1.5, 1H), 7.92 (d, *J* = 1.5, 1H); see Figure 3. ^{13}C NMR (125 MHz, $CDCl_3$): 13.8, 60.3, 117.1, 117.7, 122.7, 123.8, 124.2, 124.3, 124.4, 124.6, 124.7, 125.6, 127.9, 128.0, 128.4, 128.7, 134.0, 134.3, 136.0, 136.4, 136.8, 137.1, 137.7, 140.7, 143.1, 143.6, 152.7, 164.9; see Figure 4. MS (ESI): *m/z* 523 (M + H^+).

Poly[ethyl 6-(2,2':5',2''-terthiophen-5-yl)-1-methylene-3-phenyl-1*H*-indene-2-carboxylate] (poly-6-TT-BF3k).

A mixture of indenol **3** (318 mg, 0.588 mmol) in chloroform (450 mL) containing *p*-toluenesulfonic acid monohydrate (168 mg, 0.883 mmol) and 2,6-di-*tert*-butyl-4-methylphenol (130 mg, 0.59 mmol) was heated to reflux for 45 min. The reaction mixture was cooled to room temperature and washed in sequence with a saturated solution of sodium bicarbonate and with water. The organic layer was dried over sodium sulfate, concentrated to a volume of ca. 30 mL and purified by flash chromatography with chloroform as the eluent to obtain a solution of pure monomer 6-TT-**BF3k** in chloroform. The solution of the monomer was concentrated under reduced pressure and then dissolved again in chloroform. This procedure of dissolution/evaporation in chloroform was repeated for 5 times, while the polymerization process was followed by TLC analysis of the residues obtained after solvent evaporations. A solution of the final residue in dichloromethane (6.0 mL) was added dropwise into diethyl ether (24 mL) and the precipitate was collected by filtration and dried under reduced pressure. The yellow solid was then washed with *n*-hexane (10 mL) and dried under reduced pressure to obtain poly-6-TT-**BF3k** (143 mg, yield 47%). ^{1}H NMR (400 MHz, $CDCl_3$): see Figure 3. ^{13}C NMR (125 MHz, $CDCl_3$): see Figure 4.

References

1. A. Cappelli, V. Razzano, M. Paolino, G. Grisci, G. Giuliani, A. Donati, R. Mendichi, F. Samperi, S. Battiato, A. C. Boccia, A. Mura, G. Bongiovanni, W. Mróz and C. Botta, *Polym. Chem.* 2015, **6**, 7377-7388.

2. A. Cappelli, M. Anzini, S. Vomero, A. Donati, L. Zetta, R. Mendichi, M. Casolaro, P. Lupetti, P. Salvatici and G. Giorgi, *J. Polym. Sci. Part A: Polym. Chem*. 2005, **43**, 3289-3304.

3. A. Cappelli, S. Galeazzi, G. Giuliani, M. Anzini, A. Donati, L. Zetta, R. Mendichi, M. Aggravi, G. Giorgi, E. Paccagnini and S. Vomero, *Macromolecules*, 2007, **40**, 3005-3014.

4. A. Cappelli, F. Villafiorita Monteleone, G. Grisci, M. Paolino, V. Razzano, G. Fabio, G. Giuliani, A. Donati, R. Mendichi, A. C. Boccia, M. Pasini and C. Botta, *J. Mater. Chem.* C, 2014, **2**, 7897-7905.

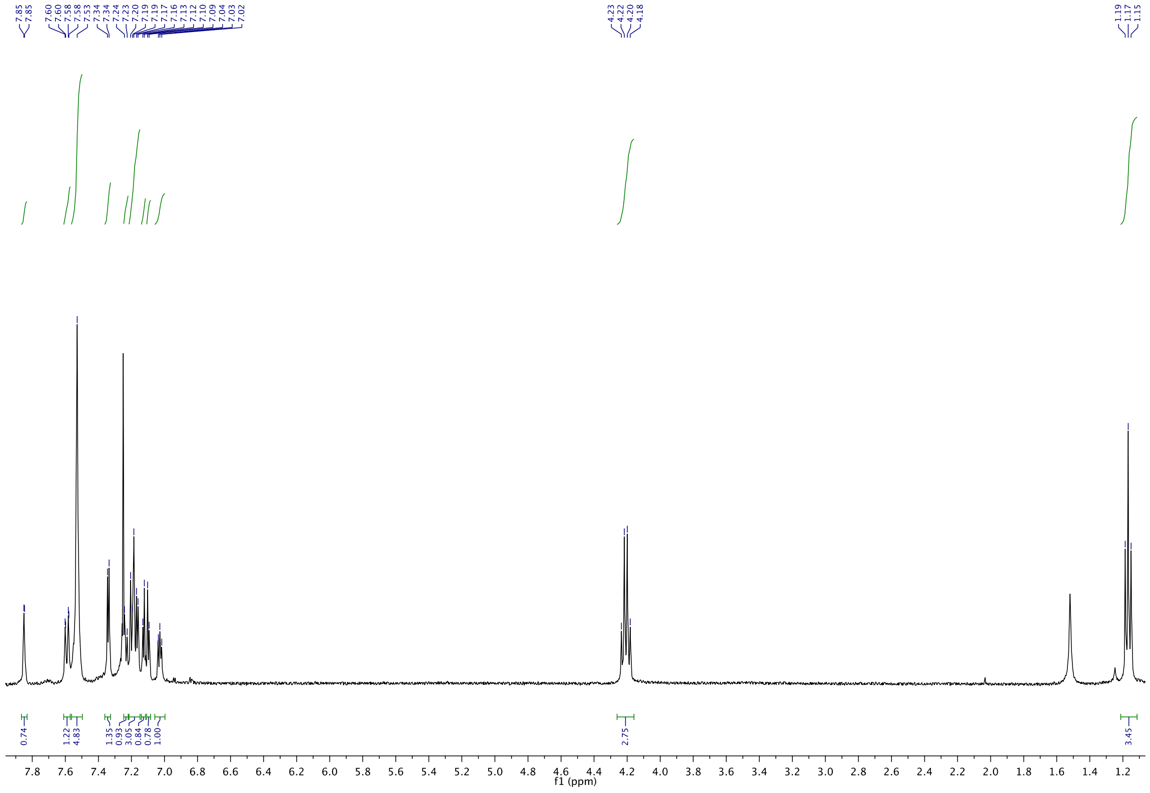

Figure ESI-3. ^{1}H NMR (400 MHz, $CDCl_3$) of ethyl 6-(2,2':5',2''-terthiophen-5-yl)-1-oxo-3-phenyl-1*H*-indene-2-carboxylate (**2**).

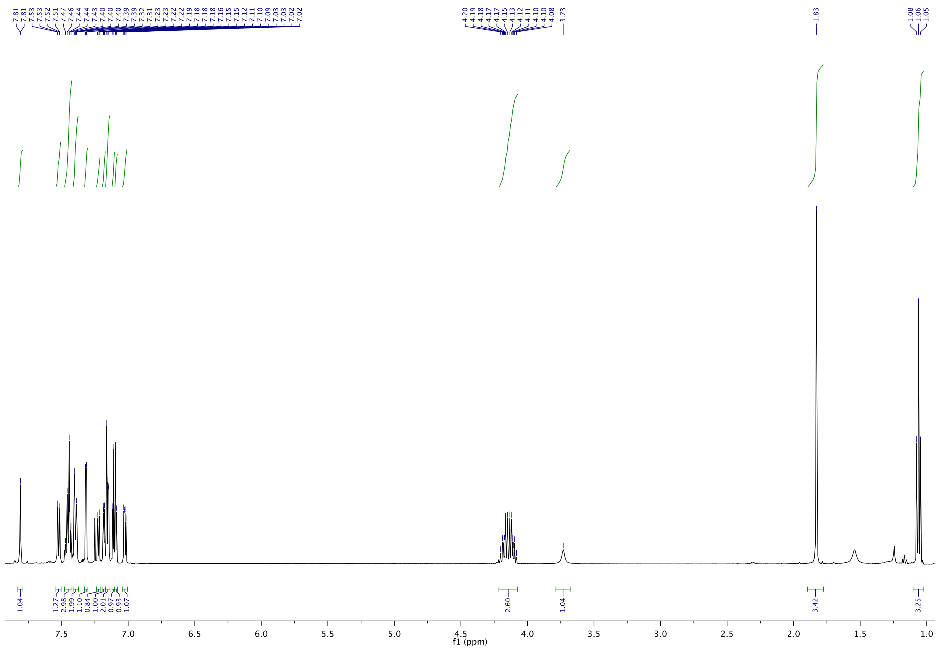

Figure ESI-4. ^{1}H NMR (500 MHz, $CDCl_3$) of ethyl 6-(2,2':5',2"-terthiophen-5-yl)-1-hydroxy-1-methyl-3-phenyl-1*H*-indene-2-carboxylate (**3**).

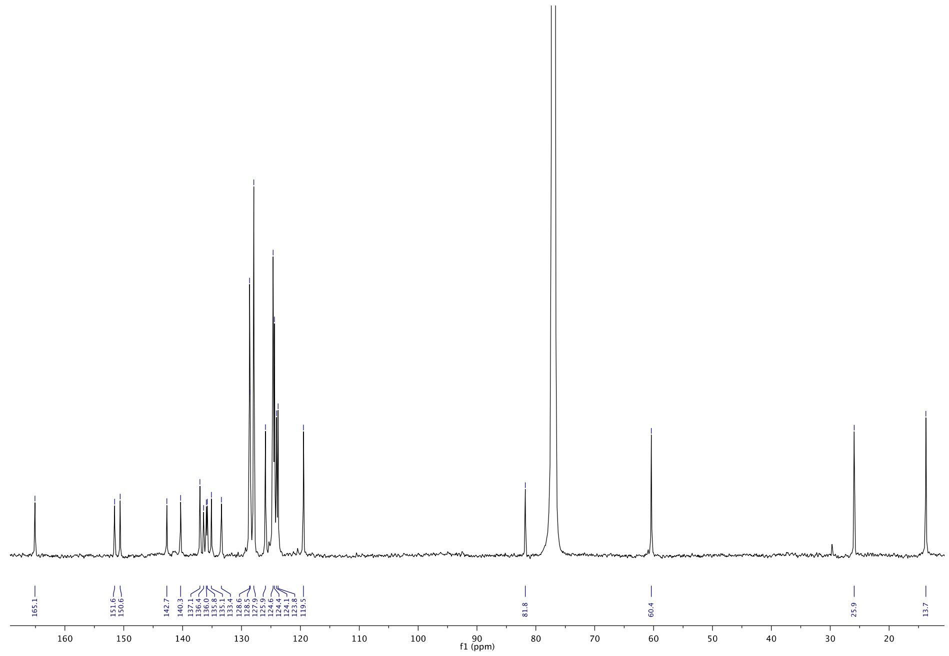

Figure ESI-5. ^{13}C NMR (125 MHz, $CDCl_3$) of ethyl 6-(2,2':5',2"-terthiophen-5-yl)-1-hydroxy-1-methyl-3-phenyl-1*H*-indene-2-carboxylate (**3**).

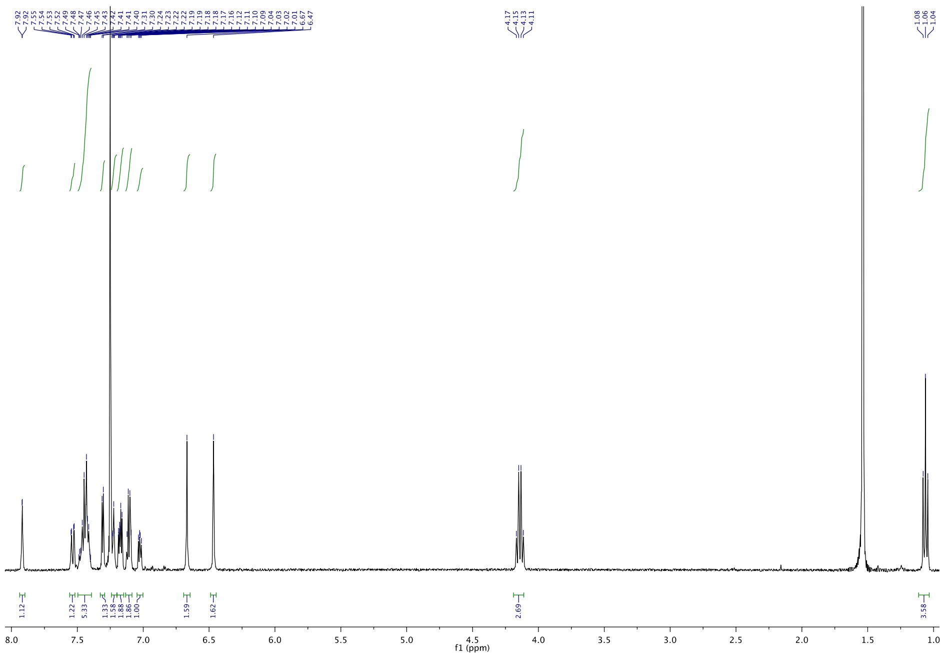

Figure-ESI-6. ^{1}H NMR (400 MHz, $CDCl_3$) of ethyl 6-(2,2':5',2"-terthiophen-5-yl)-1-methylene-3-phenyl-1*H*-indene-2-carboxylate (6-TT-**BF3k**).

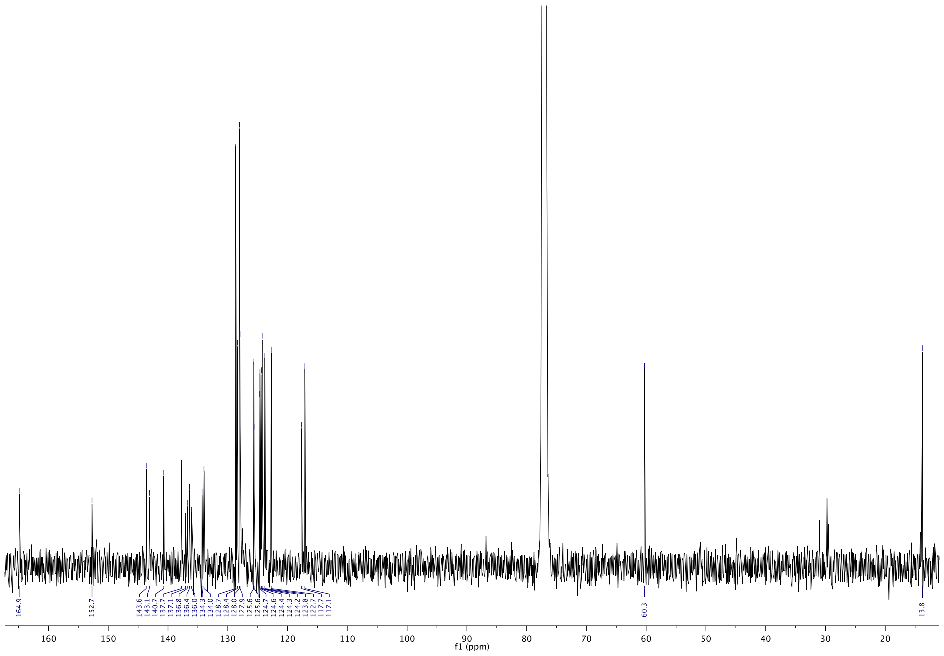

Figure ESI-7. ^{13}C NMR (125 MHz, $CDCl_3$) of ethyl 6-(2,2':5',2"-terthiophen-5-yl)-1-methylene-3-phenyl-1*H*-indene-2-carboxylate (6-TT-**BF3k**).
